# Seasonally dynamic ecosystems within offshore wind power monopile foundations support microbially influenced corrosion

**DOI:** 10.3389/fmicb.2026.1886489

**Published:** 2026-09-03

**Authors:** Nicole Adam-Beyer, Carolin Skottke, M. Schmidt, Claus-Henning Solterbeck, Emanuel Wyler, Markus Landthaler, Jana Schloesser, Mirjam Perner

**Affiliations:** 1 Geomicrobiology, GEOMAR Helmholtz Centre for Ocean Research Kiel, Kiel, Germany; 2 F&E GmbH, Kiel, Germany; 3 Benthic Biogeochemistry, GEOMAR Helmholtz Centre for Ocean Research Kiel, Kiel, Germany; 4 University of Applied Sciences Kiel, Kiel, Germany; 5 Max Delbrück Center for Molecular Medicine in the Helmholtz Association, Berlin Institute for Medical Systems Biology (BIMSB), Berlin, Germany; 6 Institute for Biology, Humboldt University of Berlin, Berlin, Germany

**Keywords:** *in situ* incubation, iron-oxidizing bacteria, MIC, microbial communities, offshore monopile, sulfate-reducing bacteria

## Abstract

**Inroduction:**

Microbially influenced corrosion (MIC) is a widespread problem in the maritime sector causingsevere economic damage. Foundation structures (monopiles) of offshore wind power systemsappear particularly prone to MIC. The hollow structures trap seawater and thus provide only limited exchange with the ambient waters. These special environmental conditions inside monopiles play a pivotal role for shaping the microbial community and respective biogeochemical processes. Currently, corrosion protection measures inside offshore monopiles only address electrochemical corrosion, likely resulting from a lack of knowledge of the microbial processes and taxa involved in MIC in these specific environments.

**Methods:**

Here we report on two *in situ* incubation experiments conducted inside a monopile structure located in the German North Sea. Mild steel (S355), as commonly used for monopiles, was incubated inside the largely enclosed structure in surface near and bottom waters for 3 and 12 months, respectively. Parallel to *in situ* incubations, temporal and spatial geochemical and microbial water column profiling was performed. Steel biofilms were analyzed with a suite of scanning electron and 3D microscopy, quantification of extracellular polymeric substances, and RNA-based 16S rRNA gene profiling, correlated with environmental parameters.

**Results and discussion:**

Geochemical water column parameters demonstrated strong seasonal vertical stratification inside the monopile. In summer months, anoxia (O_2_ = 0 μM) can develop in bottom waters and biogenic methane and highly corrosive, toxic hydrogen sulfide in bottom waters accumulated. Compared to water column samples, biofilms of the steel incubations at different depths exhibited lower microbial diversities. Microbial taxa commonly associated with MIC like Feoxidizing bacteria (e.g. *Hoeflea*), sulfate reducing bacteria (e.g. *Desulfobacteraceae*) and methanogenic archaea (e.g. *Methanococcaceae*) as well as pitting corrosion gave indications for the onset of MIC processes during the incubation of the steel coupons. In summary, the highly dynamic environmental parameters of the water column and duration of the exposure of steel structures significantly influenced the microbial community composition on steel surfaces and thus may also influence the potential for MIC-related material degradation inside monopiles that are not protected against corrosion.

## Introduction

Offshore wind power systems globally represent an essential part of decarbonization strategies and the transition to renewable energy generation, mitigating climate change. Given the higher speed and consistency of offshore winds compared to the situation onshore, offshore wind technology is a reliable and powerful source of clean energy for coastal regions (von Jouanne et al., 2025). Worldwide, growing numbers of offshore wind farms are planned, under construction or already operating. The majority of the current offshore wind turbines (especially in shallow waters up to 30 m depth) are grounded on so-called monopiles: single, cylindric piles made of steel, that are driven into the seabed (Díaz and Guedes Soares, 2020). Like other steel-based maritime infrastructure, monopiles and their respective service lifespans are strongly affected by iron corrosion processes, which are estimated to incur costs of ca. 2.5 trillion $(US) for the maritime industry worldwide (Koch, 2017). In addition to electrochemical or physical corrosion, microbially influenced corrosion (MIC) can be a significant driver, expected to account for 20%–40% of observed corrosion damages (Wolodko et al., 2018; Rao and Mulky, 2023).

The insides of offshore monopiles demonstrate special environments and habitats for MIC-associated microorganisms. The cylindric piles enclose a water body when being rammed into the seafloor and are typically characterized by a limited exchange with the ambient seawater – only possible via a limited number of water replenishment holes or cable entries. Depending on the size, number and position of these water exchange zones as well as external and resulting internal wave heights, mixing of the water column is reduced and a more or less stagnant water body can develop inside the monopile (Kesgin et al., 2024). Under such static water conditions, abiotic corrosion as well as microbial oxygen consumption (especially in the enclosed sediments) can foster deoxygenation of bottom waters. However, storm events break this stratification and lead to full mixing, including the reoxygenation of the water body again (cf. Glud, 2008; Perner et al., 2022; Skottke et al., 2025). These dynamic conditions affect the electrochemical, physical and microbially influenced corrosion of the monopile’s steel walls. During the electrochemical reaction, electrons flow from the anode to the cathode (e.g., from Fe^0^ to O_2_). By altering the electrochemical conditions at the metal-water interface, microorganisms can facilitate, initiate and/or accelerate the corrosion process, leading to corrosion rates of up to 10 mm per year (depending on material and environment) (Machuca Suarez and Polomka, 2018; Dawuda et al., 2021). Especially in its early stages, MIC is difficult to detect or predict as it occurs sporadically and is caused by phylogenetically and metabolically diverse microorganisms, influenced by differing environmental conditions (Knisz et al., 2023). The main microbial processes include biofilm formation, production of corrosive metabolites like hydrogen sulfide and nitrite (indirect MIC or MMIC, i.e., metabolite-induced corrosion), and direct electron uptake from the metal/Fe^0^ (eMIC). Microorganisms reported to catalyze MIC comprise phylogenetically and metabolically diverse groups, including sulfate-reducing bacteria (SRB), acid producers, iron oxidizers and reducers, nitrate reducers, and methanogenic archaea (Knisz et al., 2023; Xu et al., 2023). SRB are known to catalyze both eMIC and indirect MIC, the latter by producing highly corrosive hydrogen sulfide (H_2_S), essentially leading to the formation of iron sulfide (FeS). The presence and activity of SRB is commonly regarded as the main cause for MIC of iron, especially in anoxic environments (Enning and Garrelfs, 2014). It has to be noted, though, that most of the knowledge on the processes and microbial taxa involved in MIC results from laboratory incubation studies (often with model organisms) which, even if employing greatest efforts, cannot fully represent the specific environmental conditions and seed microbial communities present *in situ* (Knisz et al., 2023). MIC often occurs in the form of pitting corrosion (Xu et al., 2023). The danger here is that, even if the overall surface attack and mass change are minor, the corrosion can penetrate deeply in individual areas, thereby weakening the mechanical integrity of a steel structure without this being immediately apparent. Furthermore, the formation of hydrogen during microbial or electrochemical oxidation of Fe^0^ can lead to hydrogen-induced stress corrosion cracking, especially in an acidic and H_2_S-rich environment, which causes further material damage (Martin and Sofronis, 2022).

Although neglected in the beginning, measures against electrochemical corrosion inside offshore monopiles can meanwhile be considered “state of the art.” Two systems are frequently used, which can also be combined in the same monopile: (i) sacrificial anode cathodic protection (SACP) where an electrochemically less noble metal than monopile steel releases electrons and (ii) impressed current cathodic protection (ICCP), where an external DC power source is connected to inert anodes. In both cases, the monopile steel walls act as a cathode in the electrochemical reaction, receiving electrons (Plagemann and Momber, 2018; ISO, 2022:24656). However, no economically feasible and environmentally friendly measures are in place that specifically target the inhibition of MIC inside monopiles. In order to develop such effective MIC inhibition measures, a thorough knowledge of the microorganisms involved in the specific system as well as environmental parameters influencing the respective microbial communities is substantial. Given the restricted access to the monopiles’ interiors, sampling and investigations of occurring MIC at the actual monopile walls, and especially close to the seafloor, remains difficult (if not impossible). Previous studies aiming at characterizing corrosion occurring in the submerged parts in- or outside a monopile used steel coupons to determine corrosion rates and propose MIC taking place inside the monopile, but do not integrate changing environmental conditions or analyses of associated microbial communities (Khodabux et al., 2020; Khodabux et al., 2021).

For the present study, we aimed at filling this knowledge gap by investigating the microbial colonization of mild steel inside a monopile structure to assess potential microbially influenced corrosion in the absence of corrosion protection measures. Coupons of S355 steel (commonly used for the construction of monopiles) were incubated inside the monopile of the FINO3 research platform in the North Sea using specially designed sample holders. Incubations were conducted for 3 months (July to October 2023) at 6 m and 21 m water depth (Skottke et al., 2025) and for 12 months (October 2023 to October 2024) at 6 m, 15 m and 21 m. Water column chemistry was monitored in September and October 2023 as well as in June 2024 and October 2024 inside the foundation structure. Additionally, steel coupons from the 3-month *in situ* incubation were subjected to further laboratory cultivation under controlled oxic conditions or oxygen depleted water conditions simulating oxygen availability in the distinct *in situ* depth horizons. Water column and steel biofilm communities were analyzed using RNA-based 16S rRNA gene amplicons, which were correlated with the measured water column parameters. Further characterizations of steel biofilms and the respective matrices were performed via Scanning Electron Microscopy (SEM), 3D microscopy, energy-dispersive X-ray spectroscopy (EDS), and the analysis of extracellular polymeric substances (EPS).

## Materials and methods

### Sampling / deployment site

Water samples were taken and *in situ* incubations deployed inside the grounding structure of the research platform FINO3. FINO is the acronym for “Forschungsplattform in Nord- und Ostsee” (research platform in the North and Baltic Sea), encompassing three research platforms in the German North and Baltic Sea. FINO3 is an offshore platform located at 55°11.7’N/007°09.5’E, 80 km west of the island Sylt (Germany) in the realm of the DanTysk wind farm (Supplementary Figure S1) and is operated by F&E GmbH.^1^ It is grounded on a monopile structure, which reaches approx. 30 m deep in the underlying sandy sediments and encloses a water column of approx. 22 m. Seven oval water exchange holes of 30 × 10 cm, partly used as cable gland and enabling minor exchange with the ambient seawater, are located at 3.3 m water depth. Corrosion protection measures inside the monopile are limited to a cathodic protection system (impressed current in the upper part and sacrificial anodes at the bottom), while the outside is protected with a coating in addition to the cathodic corrosion protection.

### Sampling procedures and *in situ* measurements

**Figure 1:**
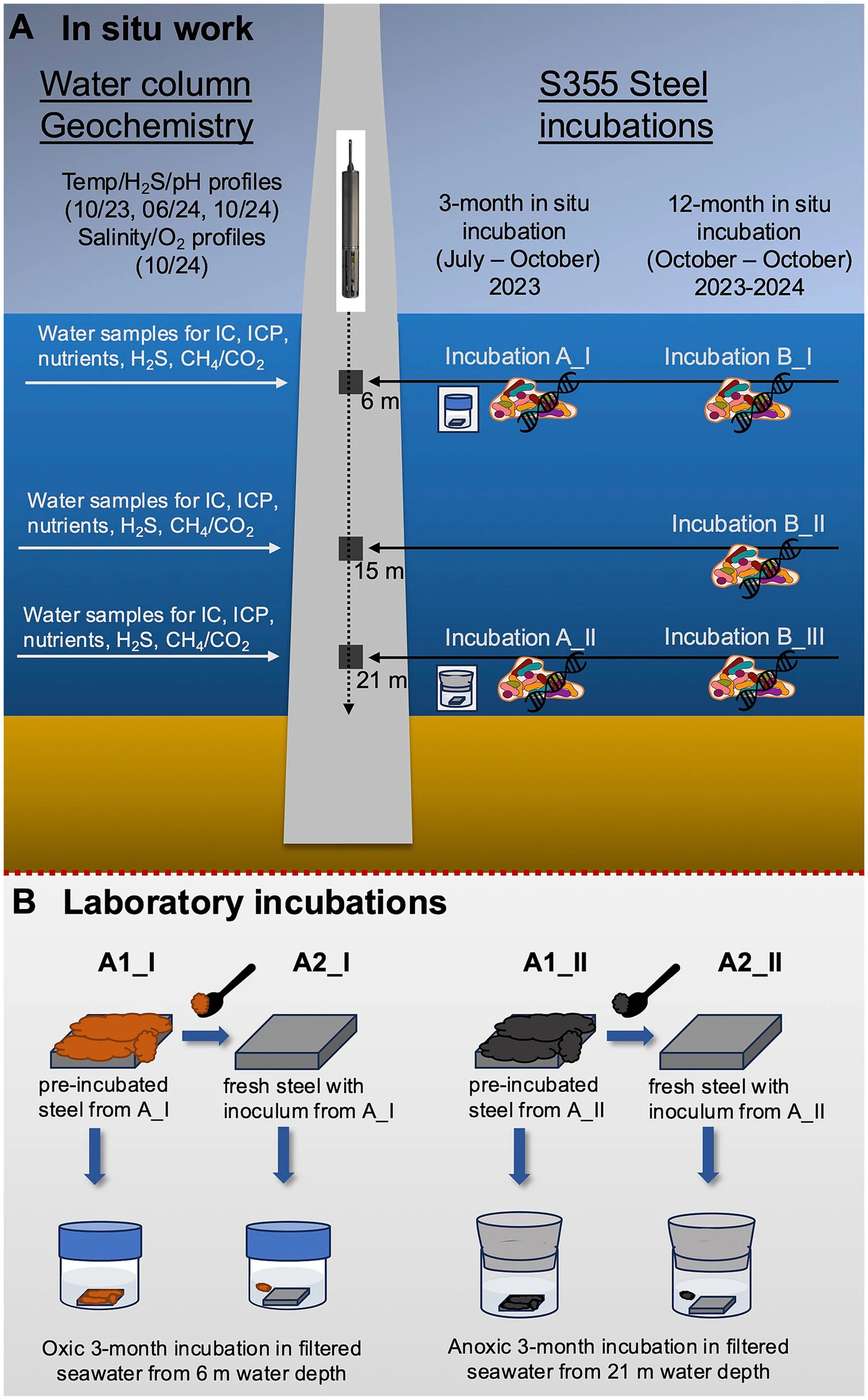
Overview of the experimental setup. *In situ* measurements and *in situ* incubations of S355 steel coupons at different water depths **(A)** were performed inside the FINO3 monopile. Subsets of *in situ* incubated steel samples were further incubated in the laboratory and also used as inoculum for lab-based incubations of freshly prepared steel coupons **(B)**.

For all deployment activities, samplings and measurements, the FINO3 platform was accessed by helicopter. Water sampling and/or *in situ* measurements of the water column inside the FINO3 monopile were carried out in September 2023 (only water sampling), October 2023 as well as June and October 2024 (water sampling and *in situ* measurements, see Figure 1). Discrete water samples were taken using a manually operated water sampler with a 2.5 L Niskin bottle at water depths of 6 m, and approx. 21 m for all time points. Additionally, in October 2023 and October 2024 samples were taken at 15 m water depth. In June 2024 an additional sampling at 11 m water depth was performed. Directly after retrieval, 200 mL samples were taken in serum bottles, closed with butyl rubber stoppers and crimp seals, for later gas analyses. In June 2024 further samples for oxygen measurements were taken in 50 mL Winkler bottles and fixed with 500 μL, each, of a MnCl_2_-solution (3.2 M) and a KI-solution (3.6 M). The remaining water of each sampling was collected in clean 1 L PP bottles and immediately subsampled in 5 mL aliquots for onshore measurements of geochemical parameters as detailed below. During the subsampling process, a filtration with syringe attachment filters (PES membrane, 0.22 μm pore size; Starlab GmbH, Hamburg, Germany) was integrated. For ICP-OES measurements, the subsamples were fixed with 1/100 volume of concentrated and distilled HNO_3_. Subsamples for H_2_S measurements were mixed with a Zn-acetate solution (final concentration of 2.5%). All samples were transported refrigerated to the home laboratory. Upon arrival, the remaining bulk water samples were filtered using Steritop filter units (0.22 μm pore size; Merck Millipore, Burlington, MA, USA). Water samples from 21 m water depth were processed under anoxic conditions using a vinyl anaerobic chamber with a N_2_: H_2_ (98%: 2%) atmosphere (Coy Laboratory Products Inc., Grass Lake, MI, USA) and subsequently stored in glass bottles sealed with butyl rubber stoppers. The filters harboring microbial cells were cut out of the filter units using sterile scalpels and stored at −70 °C for later RNA analyses, while the filtered water samples were stored at 4 °C. Subsamples for nutrient analyses and H_2_S measurements were stored frozen, while those for IC and ICP-OES measurements were kept at 4 °C until measurements were performed.

*In situ* measurements were performed using profiling probes equipped with temperature and pressure sensors as well as H_2_S (range of 10 μg/mL - 3 mg/L) and pH (H_2_S/pH/T/D probe) or oxygen and salinity sensors (CTD/O_2_ fast profiling probe, both from Analysenmesstechnik GmbH, Rostock, Germany). All sensors were calibrated by the manufacturer and calibrations of H_2_S, pH and O_2_ were checked in the home laboratory with solutions of known concentrations. While H_2_S and pH were measured from October 2023 onwards, the CTD/O_2_ probe was only used in October 2024 (Figure 1).

### Measurements of geochemical parameters and dissolved gasses in discrete water samples

Nutrients (ammonium, nitrate, nitrite, phosphate and silicate) were measured using a Quaatro Autoanalyzer (Seal Analytical GmbH, Norderstedt, Germany) according to the manufacturer’s protocols. Oxygen concentrations were determined using the standard methodology in Hansen (1999). Hydrogen sulfide in the fixed subsamples was measured spectrophotometrically according to Cline (1969). Selected cations (i.e., total iron, aluminum, manganese) dissolved in acidified (HNO_3_) subsamples were measured via inductively coupled plasma optical emission spectroscopy (Agilent 5,800 ICP-OES, Agilent Technologies, Santa Clara, Ca, USA). Sulfate, bromide and chloride concentrations were determined by ion chromatography (Eco IC, Metrohm, Herisau, Switzerland). Further analyitical details of ICP and IC measurements are described in (Micallef et al., 2026). Dissolved gases (methane and carbon dioxide) were determined via headspace-gas chromatography. After setting a headspace of 10 mL helium gas and an equilibration time of >1 h, 100 μL of headspace gas was injected into a Shimadzu gas chromatograph (GC2014, Shimadzu Corporation, Kyoto, Japan), equipped with flame ionization detector and thermal conductivity detector (carrier gas He 5.0, HayeSep™ Q 80/100 column, column length 2 m, column diameter: 1/8″). The detection limit for CH_4_ and CO_2_ was 0.1 ppm and 100 ppm, respectively. Standard gas mixtures were determined with a precision of about ±2% (RSD).

### *In situ* and ex situ incubations of S355 steel coupons

For both incubation types (*in situ* and *ex situ*) coupons of S355 mild steel with a dimension of 30 mm × 29 mm × 10 mm were used. A detailed description of the surface preparation process of the incubated samples as well as the *in situ* incubator design can be found in Skottke et al. (2025). Briefly, the samples were ground to ensure comparable surface roughness and stored under a humidity-free atmosphere to prevent corrosion. For the *in situ* incubations parallels of three coupons were arranged in incubation boxes, separated by plastic bars to avoid direct contact among the steel samples. These were then wrapped in a mesh cloth (150 μm mesh size) to prevent the colonization of macrofauna and inserted into the actual incubator, harboring a total of 9 samples in three incubation boxes per incubator. By means of a nylon rope, the incubators were deployed in the water column of the FINO3 monopile at water depths of 6 m and 21 m (incubation A) as well as 6 m, 15 m and 21 m (incubation B), where they were incubated for three (incubation A) and 12 months (incubation B), respectively (Figure 1). At the end of the incubation period, the incubators were recovered and the inner boxes (including the mesh cover) were removed from the incubator. For transportation to the home laboratory the samples were stored in plastic bags filled with water retrieved in the monopile at the respective water depth and refrigerated. Processing of the incubated steel coupons in the laboratory was started within 4–6 h after removal from the monopile. After a visual inspection, the coupons were separated for the different subsequent analyses and in case of incubation A further ex situ incubations (see Supplementary Table S1 for an overview of the analyses performed on individual samples). All samples incubated at 21 m water depth were exclusively processed under anoxic conditions using the vinyl anaerobic chamber. Corrosion products and attached biofilm material was scraped off and frozen at −70 °C for later RNA extraction and 16S metabarcoding of at least 2 steel coupons per incubator. Furthermore, scraped off material was fixed in a 2.5% glutaraldehyde solution (in 1x Phosphate-buffered saline (PBS)) and incubated at 4 °C for later scanning electron microscopy (SEM).

Two setups of ex situ incubations were run in duplicates under oxic and anoxic conditions, respectively (Figure 1): one with the previously *in situ* incubated steel coupons (incubations A1_I / A1_II) and one with freshly prepared steel coupons, inoculated with corrosion products and the attached biofilms scraped off from coupons from incubation A (incubations A2_I / A2_II). All of the ex situ incubations were carried out in 250 mL wide mouth glass bottles filled with 100 mL of filtered seawater retrieved from the inside of the FINO3 monopile during sample recovery of incubation A. For the oxic incubations (A1_I / A2_I) sample material from the 6 m *in situ* incubation was used and the culture bottles were closed with membrane venting screw caps to ensure ventilation during the incubation. The anoxic incubations (A1_II / A2_II) were set up in the anaerobic chamber and sealed with rubber stoppers of 5 cm thickness to prevent the introduction of oxygen. All lab incubations were incubated at 16 °C without shaking for 3 months. At the end of the incubation period, corrosion products and attached biofilm material was scraped off of the steel coupons and fixed for RNA extractions as described for the *in situ* incubations.

### RNA extraction and 16S metabarcoding of Bacteria and Archaea

RNA was extracted from corrosion products and attached biofilms from *in situ* and ex situ incubations as well as from filters of the seawater sampling using the ZymoBIOMICS RNA Miniprep kit (Zymo Research, Irvine, Ca, USA) according to the manufacturer’s recommendations. The optimized lysis protocol was used in combination with a BioSpec Mini-BeadBeater-16 (BioSpec Products Inc., Bartlesville, OK, USA). The DNA-free RNA was transcribed into cDNA using the SuperScript™ VILO™ cDNA synhesis kit (ThermoFisher Scientific, Waltham, MA, USA), followed by cDNA purification with the clean and concentrator-5 purification kit (Zymo Research) according to the manufacturer’s cDNA protocols. Amplicons of the bacterial V3-V4 regions and archaeal V4-V5 regions of 16S rRNA genes were generated using the Bact_341F/805R, Arch_519F/915R and Arch 524F/958R primer pairs as described in Adam et al. (2019) and Adam-Beyer et al. (2025). Purified amplicons were indexed using Nextera XT v2 index kits (Illumina Inc., San Diego, Ca, USA) according to the manufacturer’s instructions and (following another purification) sequenced in equimolar pools using a 2×300 bp paired-end run on Illumina’s NextSeq 2000 platform.

### Sequence processing and statistical analyses

Processing of demultiplexed raw reads was performed using the Qiime2 environment (Bolyen et al., 2019) as previously described (Adam-Beyer et al., 2023; Adam-Beyer et al., 2025). Briefly, primer sequences were removed and the individual raw sequences were trimmed to 270 nucleotides, each, before being merged using the dada2-plugin (Callahan et al., 2016). Taxonomic assignments were performed using a pretrained classifier based on SILVA database release 138.2 (Quast et al., 2013) and the feature-classifier plugin (Bokulich et al., 2018). Contaminating sequences (i.e., eukaryotic and chloroplast sequences as well as bacterial sequences in the archaeal data set and vice versa) were removed before calculating phylogenetic trees using the “align-to-tree-mafft-fasttree” pipeline (Price et al., 2010). Statistical analyses and plot generation (relative abundance calculations, alpha diversity (Shannon metric), Principle Coordinate analyses (PCoA) as well as Spearman correlations with environmental parameters) were performed using the microeco package release 1.7.1 (Liu C. et al., 2021) in R (version 4.4.1; https://www.r-project.org/). Prior to PCoA and correlation analyses, the bacterial and archaeal datasets were rarefied to 5,000 and 2,000 sequences, respectively. PCoA was performed based on weighted and unweighted unifrac distance matrices. For Spearman correlations environmental parameters at the respective time point of sample recovery were used and water samples and steel biofilms were analyzed separately.

### Extraction and analyses of extracellular polymeric substances

For the extraction of extracellular polymeric substances corrosion material (including the biofilm material) was scraped off of two *in situ* incubated steel coupons per incubation depth. Each sample was suspended in sterile 0.14 M NaCl solution [ratio 1:16 (w/v)] and homogenized by vortexing at full speed for 30 min, followed by a centrifugation at 4,500 g and 4 °C for 20 min. The resulting supernatant was filtered twice using cellulose-acetate filters with a pore size of 0.22 μm to remove residual microbial cells and the EPS extracts were finally stored at −20 °C.

Neutral and acidic polysaccharides were measured using a colorimetric method according to DuBois et al. (1956) using alginate and dextran for the standard curves. Uronic acids were measured with a standard of D-glucuronic acid as described in Filisetti-Cozzi and Carpita (1991). For the determination of lipids 0.5 mL of EPS extract were mixed with an equal volume of n-hexane and incubated at room temperature under vigorous shaking for 30 min. The reaction mixtures were centrifuged for 90 s at 12,000 *g*, whereafter the upper phase (containing lipids and hexane) was transferred into pre-weighed glass vials. The organic solvent was removed by gassing the open vials with pure nitrogen. Finally, the lipid content was determined by weighing the dried samples. Protein contents were determined using the Roti-Quant Bradford Assay according to the manufacturer’s protocol for the microtiter scale and with bovine serum albumin (BSA) as a standard.

### Scanning electron and 3D microscopy

After 48 h of incubation in the glutaraldehyde-solution, the fixed corrosion products of *in situ* incubated steel coupons were washed, desalted and dried. For this purpose, the samples were centrifuged at 3,000 *g* (4 °C) for 1 min, the supernatant removed and the resulting pellet gently resuspended in 10 volumes of a 1xPBS / MilliQ (75% / 25%), followed by an incubation at room temperature for 5 min. This washing step was repeated with decreasing salt (1xPBS) percentages of 50%, 25%, and 10%, followed by pure MilliQ. Subsequently, the samples were subjected to dehydration by washing with a graded series of Ethanol / MilliQ solutions (10%, 25%, 50%, 75%, 95%, and 100% of Ethanol). To complete the dehydration, the EtOH was removed and the sample was resuspended in a mixture of Ethanol and Hexamethyldisilazane (HMDS) at a 1:1 ratio. After an incubation at room temperature for 10 min, the samples were centrifuged for 1 min at 1,900 g (4 °C) and the supernatant removed. An incubation for 10 min in 100% HMDS followed, which was repeated two more times (with centrifugation in between). Finally, approx. 20 μL of the suspension were pipetted onto glass slides prepared with a coating of poly-l-lysine and air-dried overnight. SEM was performed using a FE-SEM Zeiss Ultra Plus (Carl Zeiss AG, Oberkochen, Germany). Since the fixed biofilm is not electrically conductive, the samples were sputtered with platinum using an EMITECH K575X sputter coater prior to the SEM analysis. When examining samples using SEM, a working distance of approximately 8 mm was targeted while the applied acceleration voltage was adjusted on the analysis and imaging tasks. For increased surface sensitivity the voltage was decreased down to 3 kV. For characterizations of the corroded steel surfaces, non-fixed but dried steel coupons were analyzed using SEM and EDS to determine their respective elemental compositions. Subsequent to these analyses, the corrosion products on the steel coupons were removed in accordance with DIN EN ISO 8407. The remaining surface was examined again via SEM and additional characterization of the remaining corroded surfaces was conducted using 3D measurements. These were performed with a Leica DCM8 microscope operating in focus-variation mode, and the resulting data were analyzed using the Leica Map Premium software.

## Results

The FINO3 research platform is located on the outskirts of German offshore wind farms in the North Sea. The hollow foundation structure consists of S355 steel and has seven water exchange holes the size of 30 cm × 10 cm, located 3.3 m below sea level, some of which are covered with marine fauna. Water column properties inside the monopile were combined with molecular analyses and cultivation experiments using both *in situ* and laboratory incubations with S355 steel coupons (Figure 1).

### Seasonal stratification of the water column inside the monopile

**Figure 2:**
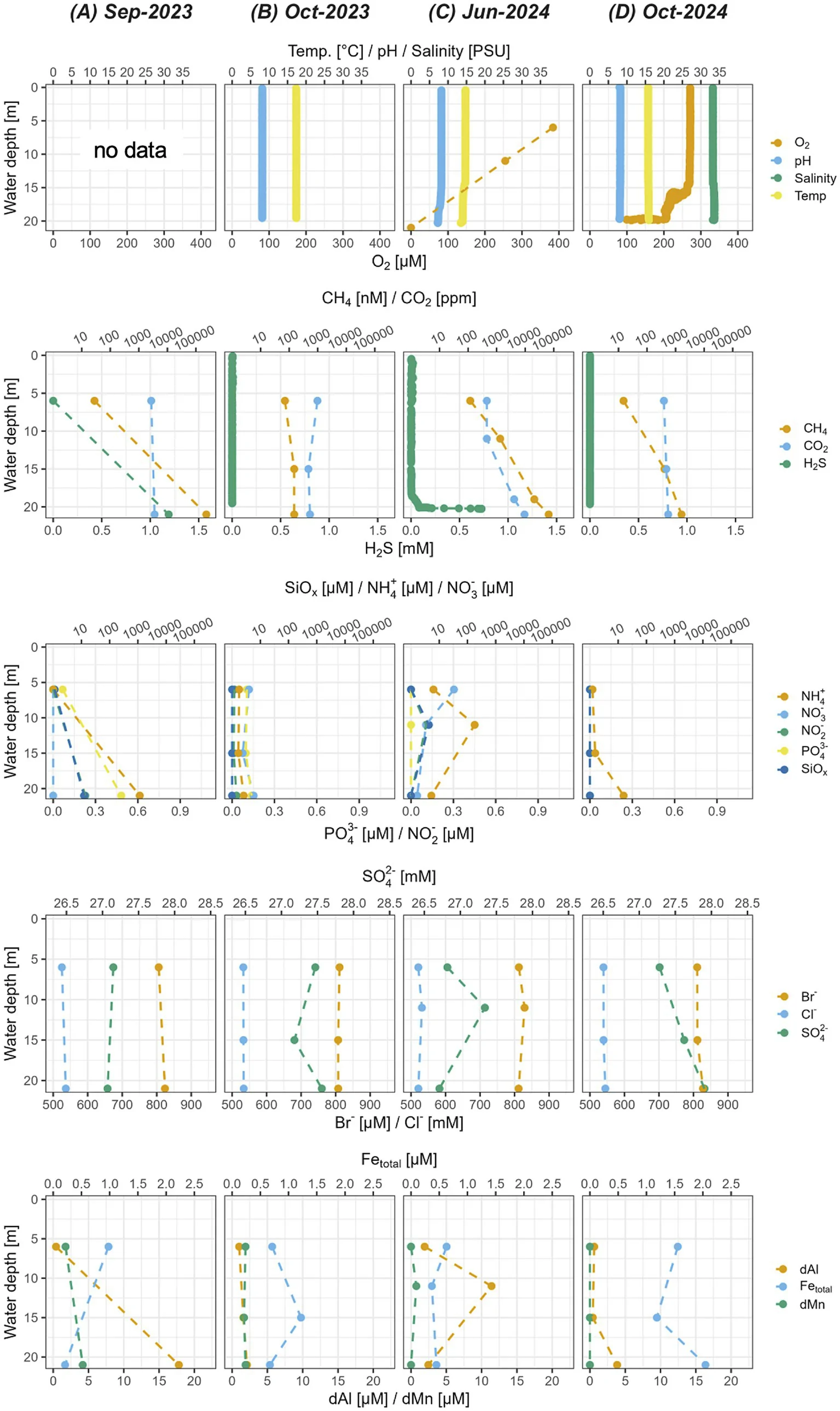
Water column geochemistry measured inside the FINO3 monopile in September 2023 **(A)**, October 2023 **(B)**, June 2024 **(C)**, and October 2024 **(D)**. Dashed lines were used to connect individual measurements of discrete water samples.

Water column properties were determined at four time points during summer/fall in 2023 and 2024. Seasonal stratification inside the FINO3 monopile was observed for September 2023, June 2024 and October 2024, while October 2023 suggested a good ventilation of deeper waters (Figures 2A–D).

In September 2023 bottom waters inside the monopile (1 m above the ground, ca. 21 m water depth) contained 1.2 mM H_2_S, 232 μM CH_4_ and 35,858 ppm CO_2_ indicating that the stratification had developed over a longer time period. Additionally, ~18 μM of aluminum had accumulated in bottom waters, most likely originating from the sacrificial anodes installed inside the FINO3 monopile. This concentration represents the solubility limit for oxidized aluminum under the (calculated) pH and temperature conditions present at the time of sampling. The overall highest concentrations of nutrients (with the exception of nitrate) were also measured in bottom waters of September 2023, with ammonium reaching 1.2 mM (Figure 2A). Only 4 weeks later, following a major storm event in the North Sea, the water stratification was completely broken inside the foundation structure as demonstrated in the geochemical profiles (Figure 2B): no thermo- or chemocline were observed and H_2_S levels in bottom waters were below the detection limit of ~0.3 μM. However, at 15 m water depth peaks of total Fe and CH_4_ (1.22 μM and 150 nM, respectively) were observed. These peaks likely result from (bio)chemical processes inside the monopile: in the course of (bio)chemical Fe^0^-oxidation hydrogen is released, which in turn fuels the biogenic formation of CH_4_ by methanogens (Xu et al., 2023). While the nutrient concentrations in the bottom waters were predominantly lower in October than in September 2023, those measured at 6 m water depth were up to 21 times higher, reflecting the typical replenishment of nutrients in surface water layers of the North Sea (Brockmann and Kattner, 1997; Brockmann and Topcu, 2002).

Vertical profiles in June 2024 exhibited a thermo- and chemocline at around 15 m water depth. While oxygen concentrations decreased with depth (0 μM oxygen measured at 1 m above the seafloor), CH_4_ and CO_2_ concentrations increased with depth, with maxima of ~66 μM and 9,466 ppm, respectively (Figure 2C). At 21 m water depth the H_2_S concentration was 0.7 mM. Phosphate remained below detection limit throughout the water column. However, the highest nitrate concentration (33 μM) of all sampling points was measured at 6 m water depth in June 2024 (Figure 2). All other measured nutrients peaked at 11 m water depth with up to 180 μM ammonium and 0.1 μM nitrite (Figure 2C).

In October 2024 the water column stratification was still visible in the vertical profiles, though noticeably less pronounced than in June 2024 (Figures 2C,D). Although a decrease of the oxygen concentration was observed below 15 m water depth, anoxic conditions no longer prevailed in the bottom water and also the H_2_S accumulated during the summer had been completely consumed (Figure 2D). CH_4_ and CO_2_ concentrations still showed a depth-related increase, albeit weaker than in June 2024 (up to 1.6 μM and 551 ppm, respectively). Furthermore, the highest concentrations of total Fe (up to 2.05 μM) and sulfate (up to 27.9 mM) were measured in discrete water samples from October 2024 (Figure 2D). In contrast to October 2023, in October 2024 all nutrients except for ammonium – peaking with 15 μM in the bottom water - were completely depleted.

### Visual observations, microscopy and EDS-analyses of *in situ* incubations of S355 steel coupons

**Figure 3:**
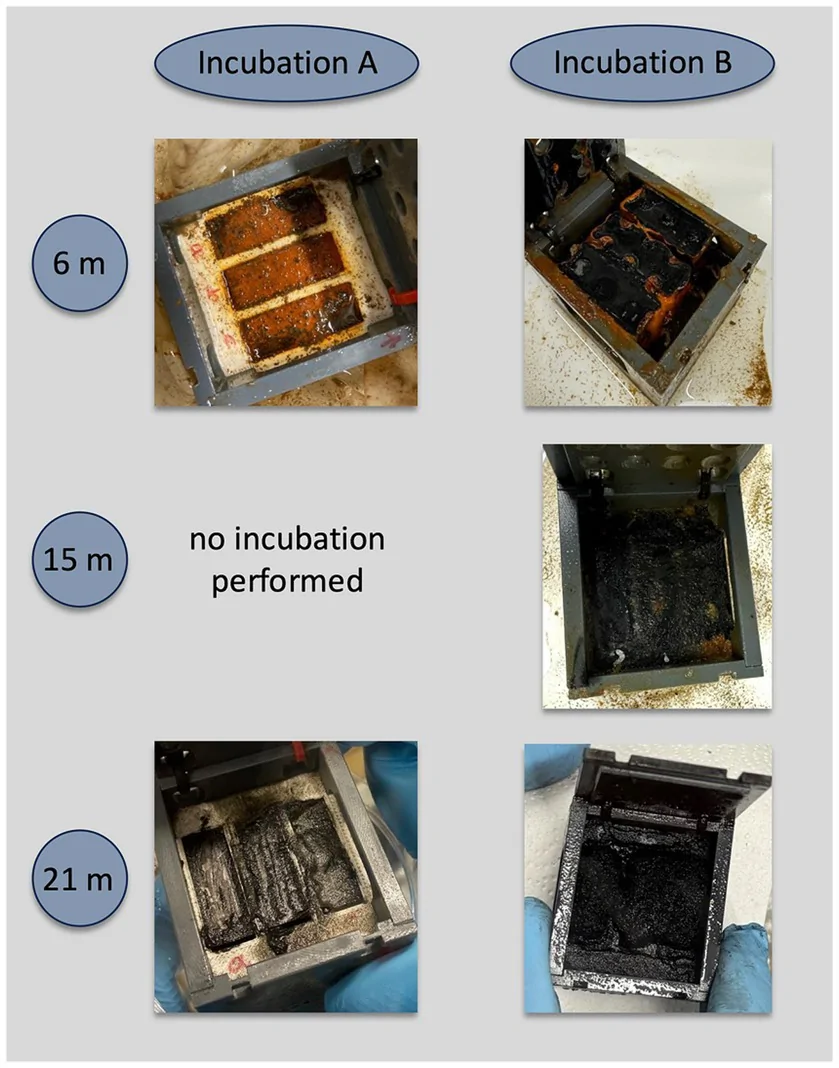
*In situ* incubated steel samples during the subsampling process after 3 months (incubation A) and 12 months (incubation B) inside the FINO 3 monopile. Incubation A was only performed at two different water depths (6 m and 21 m), while incubation B comprised an additional incubation set at 15 m water depth.

After the recovery, the *in situ* incubated S355 steel coupons of both incubations (A and B) showed clear signs of corrosion. In the 3-month incubation (incubation A), the steel coupons incubated at 6 m water depth were covered in reddish brown corrosion products indicative of iron oxides and oxic corrosion processes. Those incubated at 21 m water depth wore black corrosion products and emitted a characteristic smell of sulfide, indicating that iron sulfides were formed under anoxic conditions during the incubation. Overall, the steel coupons of incubation B (incubated for 12 months) were characterized by a notably thicker, yet softer layer of corrosion products (and biofilm) compared to those of incubation A (Figure 3). In addition to the reddish-brown iron oxides, the 6 m and 15 m steel samples here also exhibited black corrosion products (Figure 3). Again, the steel coupons incubated at 21 m water depth showed no obvious signs of iron oxides but were solely covered in black corrosion products (Figure 3) and a smell of sulfides.

**Figure 4:**
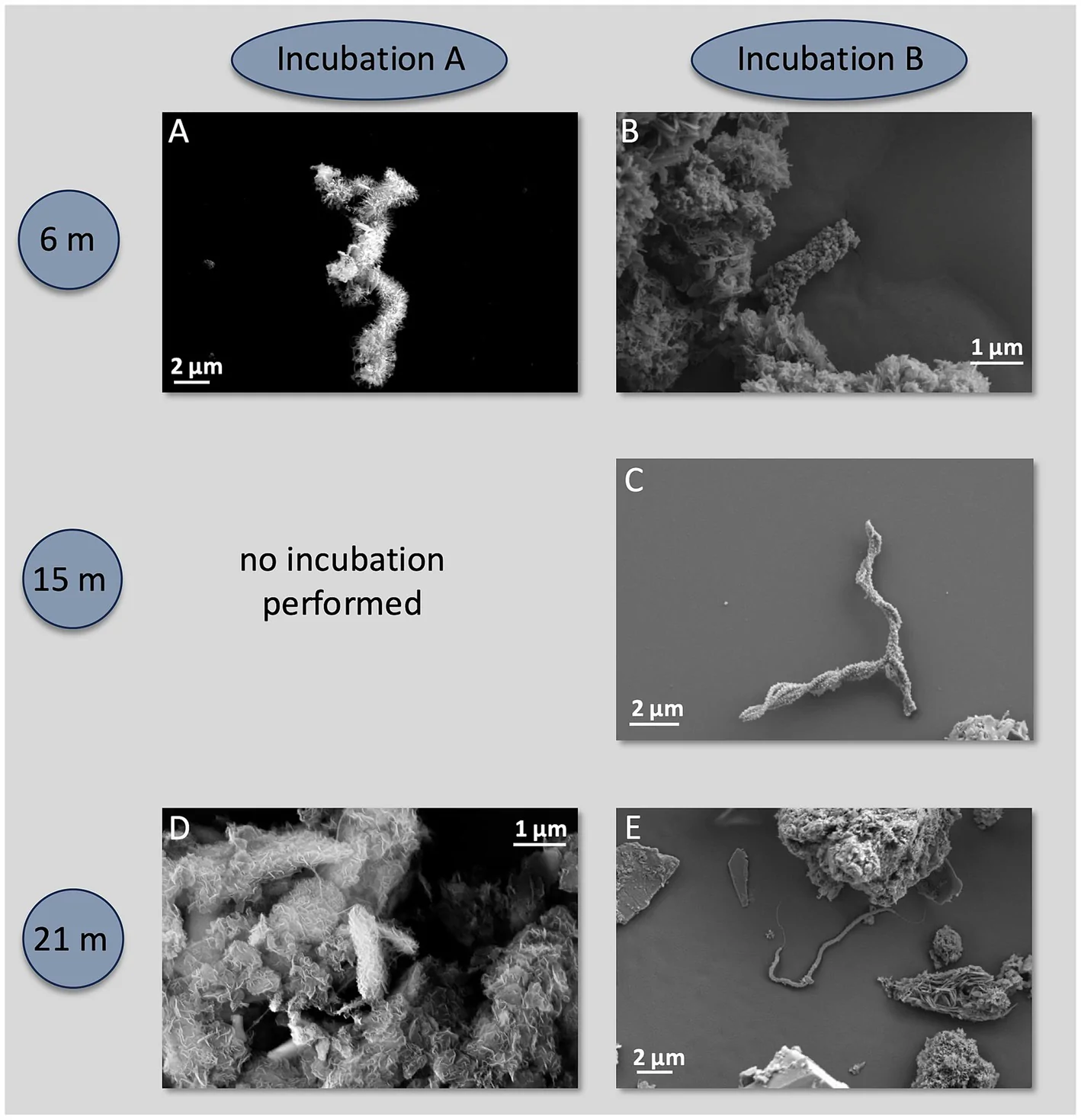
Scanning electron microscopy (SEM) images of fixed corrosion products from the 3-month *in situ* incubation A **(A,D)** and the 12-month *in situ* incubation B **(B, C, E)**.

SEM imaging of corroded steel coupon surfaces (corrosion products not removed) showed sheaths and twisted stalks in all analyzed samples of the 6 m and 15 m incubations - regardless of the incubation time (Figures 4A,C; Supplementary Figure S5). These characteristic structures are typically produced by Fe-oxidizing bacteria to avoid encrustation of the cell by crystalline iron oxyhydroxides formed during Fe-oxidation (Chan et al., 2016; Vigliaturo et al., 2020). In the 21 m incubation, sheet-shaped depositions could indicate the presence of FeS and colonization with SRB (c.f. Figure 4; Supplementary Figure S5; Akhtar et al., 2012; Tamisier et al., 2023), especially given the sulfidic odor of the steel coupons. Corresponding to these observations, exemplary EDS measurements to characterize the elemental composition of the corrosion products demonstrated an increase in sulfur contents with incubation depth (Table 1). The highest proportion of sulfur [≈ 18% (w/w)] was measured for the analyzed steel coupon of the 21 m incubation A. In this 3-month incubation no sulfur was detected in the corrosion products of steel incubated at 6 m water depth, while it constituted 1–2% of the corroded surface of the analyzed steel coupon of the 6 m incubation B. In all analyzed samples high proportions of Fe [up to 66.6% (w/w)] and oxygen [up to 31.1% (w/w)] were measured (Table 1). For the 21 m incubations (exposed to anoxic bottom water at the time of sampling), the detected oxygen most likely represents a result from sample handling for SEM and EDS analyses, where samples could not be permanently stored and handled under anoxic conditions. Other elements were mostly detected in minor proportions and reflected steel components, putative biomass and seawater components or the platinum used for sputtering the steel coupons prior to SEM/EDS analyses.

**Table 1:** Elemental compositions of corrosion products on *in situ* incubated S355 mild steel coupons, determined by EDS measurements.

Incubation	Incubation depth	Sample	Fe	O	S	Al	Ca	C	Cl	Mg	Mn	Pt	K	Si	Na
A	6 m	A_I_2b	≈ 66.6	≈ 31.1	0	0	0	<2.0	0	<1.0	0	0	0	0	<1.0
	21 m	A_II_1a	≈ 49.9	≈ 20.4	≈ 18.0	≈ 2.1	0	≈ 3.2	<1.0	<2.0	0	0	0	<2.0	≈ 2.3
B	6 m	B_I_2b	≈ 49.1	≈ 24.9	<2.0	0	0	0	≈ 10.1	0	<2.0	≈ 7.7	0	0	≈ 4.7
	15 m	B_II_2b	≈ 53.8	≈ 26.6	≈ 6.3	0	<2.0	0	≈ 2.0	<2.0	<1.0	≈ 5.9	0	<1.0	<2.0
	21 m	B_III_2b	≈ 35.0	≈ 26.0	≈ 11.7	<2.0	<1.0	0	<1.0	≈ 2.7	<1.0	≈ 6.1	<1.0	≈ 14.2	<2.0

Relative element contents are shown in % (w/w).

**Figure 5:**
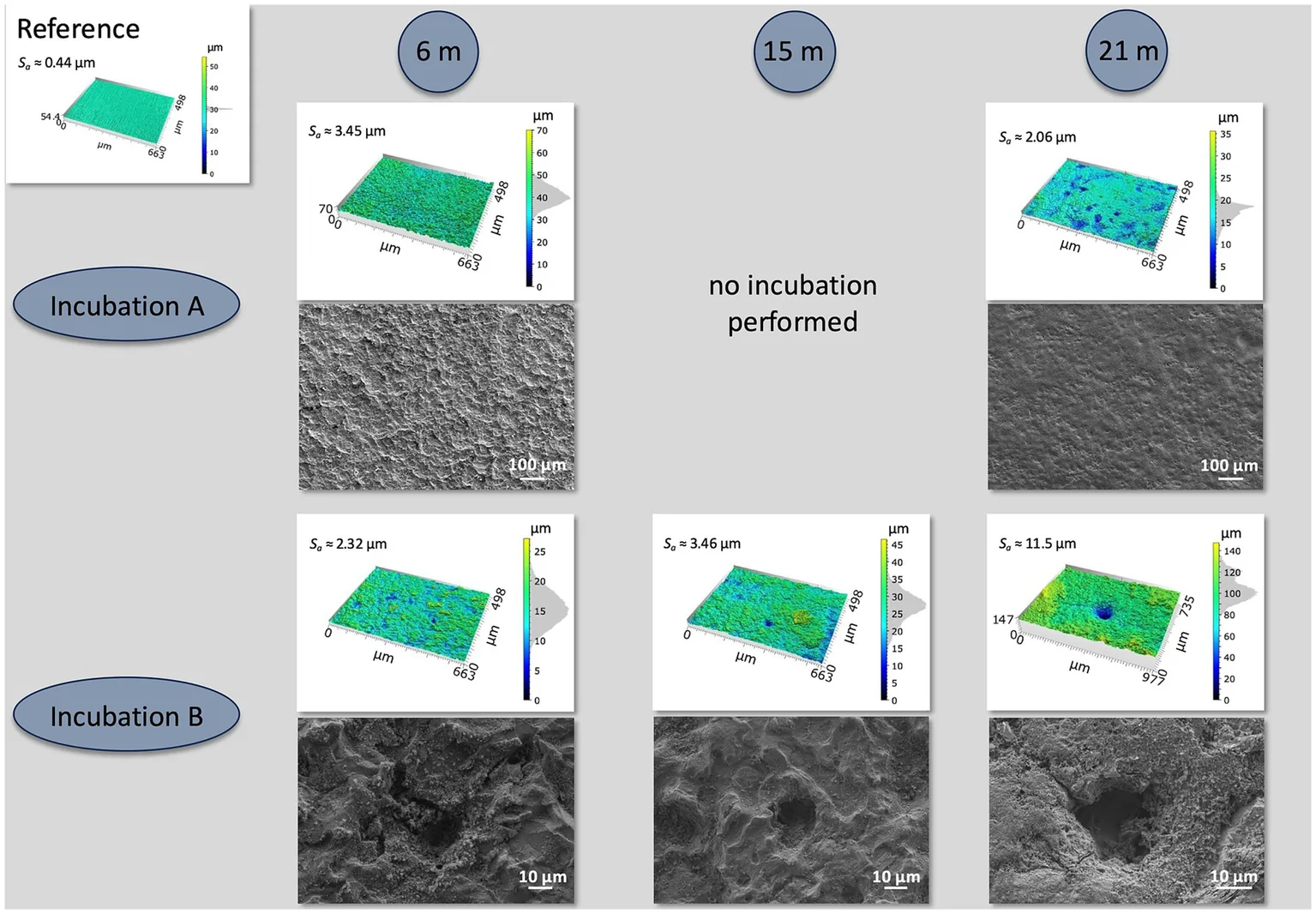
Exemplary 3D microscopy and SEM images of incubated steel coupons after the removal of corrosion products. As a reference, a 3D scan of a freshly prepared (uncorroded) steel coupon is displayed. *S*_a_ values represent the arithmetic mean height and serve as a measure of the surface roughness. SEM images of incubation B show pitting.

After SEM/EDS analyses and the removal of corrosion products, 3D-microscopy of the cleaned steel coupons revealed alterations in the surface roughness for all inspected samples of incubation A and B (Figure 5). Compared to the starting conditions, the surface roughness (as indicated by the *S_a_* value) increased 4.7- fold (21 m incubation A) to 26.1-fold (21 m incubation B, see Figure 5). In the 3-month incubation A, this effect was more prominent at 6 m incubation depth than at 21 m. Yet, no pitting was apparent for the inspected steel coupons. In contrast, steel coupons of incubation B exhibited pitting at all incubation depths with the greatest pit depths (and overall surface roughness) measured for the sample incubated at 21 m water depth (103 μm, see Figure 5). Thus, the differing incubation periods led to opposing trends in the corrosion patterns of exposed steel coupons.

### Diversity of water column and steel biofilm communities

In order to identify potential key players for MIC inside monopiles of offshore wind turbines in the North Sea we performed two *in situ* incubations inside the monopile of the FINO3 platform, of which one was further cultivated in the laboratory.

**Figure 6:**
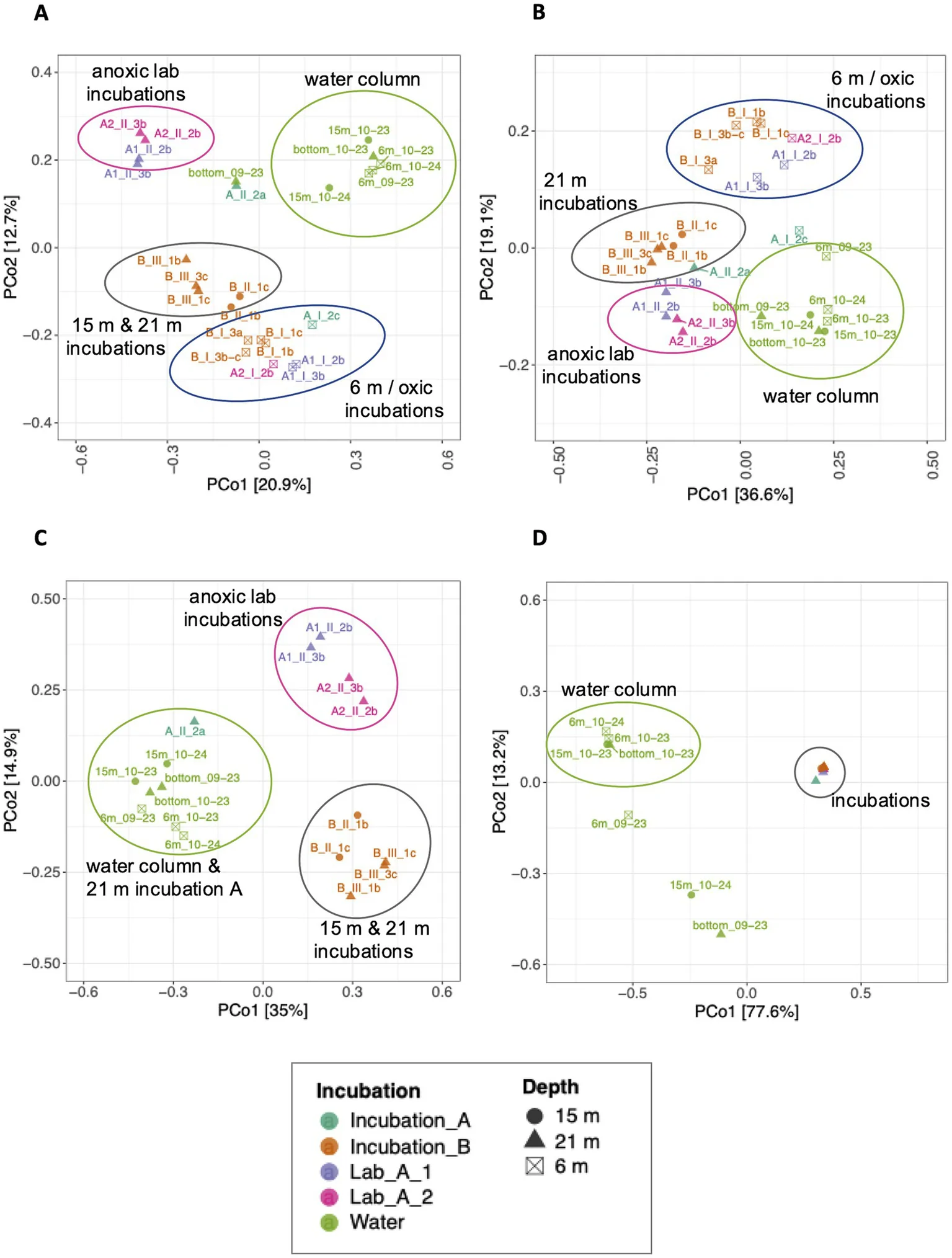
PCoA plots of Bacteria **(A,B)** and Archaea **(C,D)** based on unweighted **(A,C)** and weighted **(B,D)** Unifrac distance calculations. Clusters were drawn manually.

Analyses of 16S rRNA gene amplicons from reverse transcribed RNA of water samples and steel biofilms revealed distinct microbial communities with differing alpha- and beta-diversities across the sample types and water depths (Supplementary Figure S2; Figure 6). Compared to the water samples, the alpha-diversities of bacterial and archaeal communities based on amplicon sequence variants (ASVs) from both the laboratory and *in situ* incubations were significantly lower. While the Shannon indices for the bacterial communities were similar between the *in situ* and laboratory incubations, a further reduction of the archaeal diversity was observed for the ex situ incubations (Supplementary Figure S2). Principal coordinates analyses (PCoA) based on weighted and unweighted Unifrac distances showed a certain clustering of both the bacterial and archaeal communities according to sample type, incubation type and incubation period as well as water depth (Figure 6). The bacterial water column communities of 6 m and 15 m water depth formed a cluster exhibiting no clear differences with respect to the sampling month but a great distance to the *in situ* incubated steel communities. The 21 m water sample from September 2023 clustered with the 21 m steel biofilm from incubation A, separated from the water column cluster along coordinate 1 (in the unweighted analysis) (Figure 6A). Further along this coordinate a third cluster was observed consisting of the anoxic lab incubations of the 21 m steel biofilms (incubation A1_II and A2_II). The remaining steel biofilm samples were separated from the lab incubations and water samples along coordinate 2, forming two closer related clusters. One consisted of the 15 and 21 m steel biofilms of incubation B, the other of the 6 m *in situ* and ex situ incubated steel biofilms of both incubation periods (Figure 6A). In the weighted analysis, taking into account the relative abundances of observed ASVs, the clusters were overall less widely scattered across the two coordinates (Figure 6B). However, the 6 m steel biofilm sample cluster (excluding incubation A) exhibited a greater distance to the 15 and 21 m *in situ* cluster, which also includes the 21 m sample of incubation A. Furthermore, in the weighted analysis the 6 m water sample from September 2023 as well as the 6 m steel biofilm of incubation A were positioned in a greater distance to the other water and steel biofilm samples, respectively (Figure 6B). Due to low read numbers all of the archaeal steel biofilm samples incubated *in situ* at 6 m water depth were removed during the rarefication for the PCoA (Figures 6C,D). While the 21 m biofilm of incubation A clustered together with all of the water samples, the remaining *in situ* incubated steel biofilms (incubation B) formed a separate cluster, positioned at a greater distance to the other samples in the unweighted analysis of archaeal sequences (Figure 6C). In the weighted analysis for Archaea most of the water samples formed a tight cluster with the exception of both samples from September 2023 and the 15 m water sample from October 2024, which were scattered mostly along coordinate 2. All of the remaining archaeal samples (lab and *in situ* incubations) grouped together very closely in the weighted PCoA (Figure 6D), indicating a strong enrichment of individual groups of the archaeal communities during the incubation.

### Community compositions of water column and steel biofilm communities – Bacteria

#### Water column

**Figure 7:**
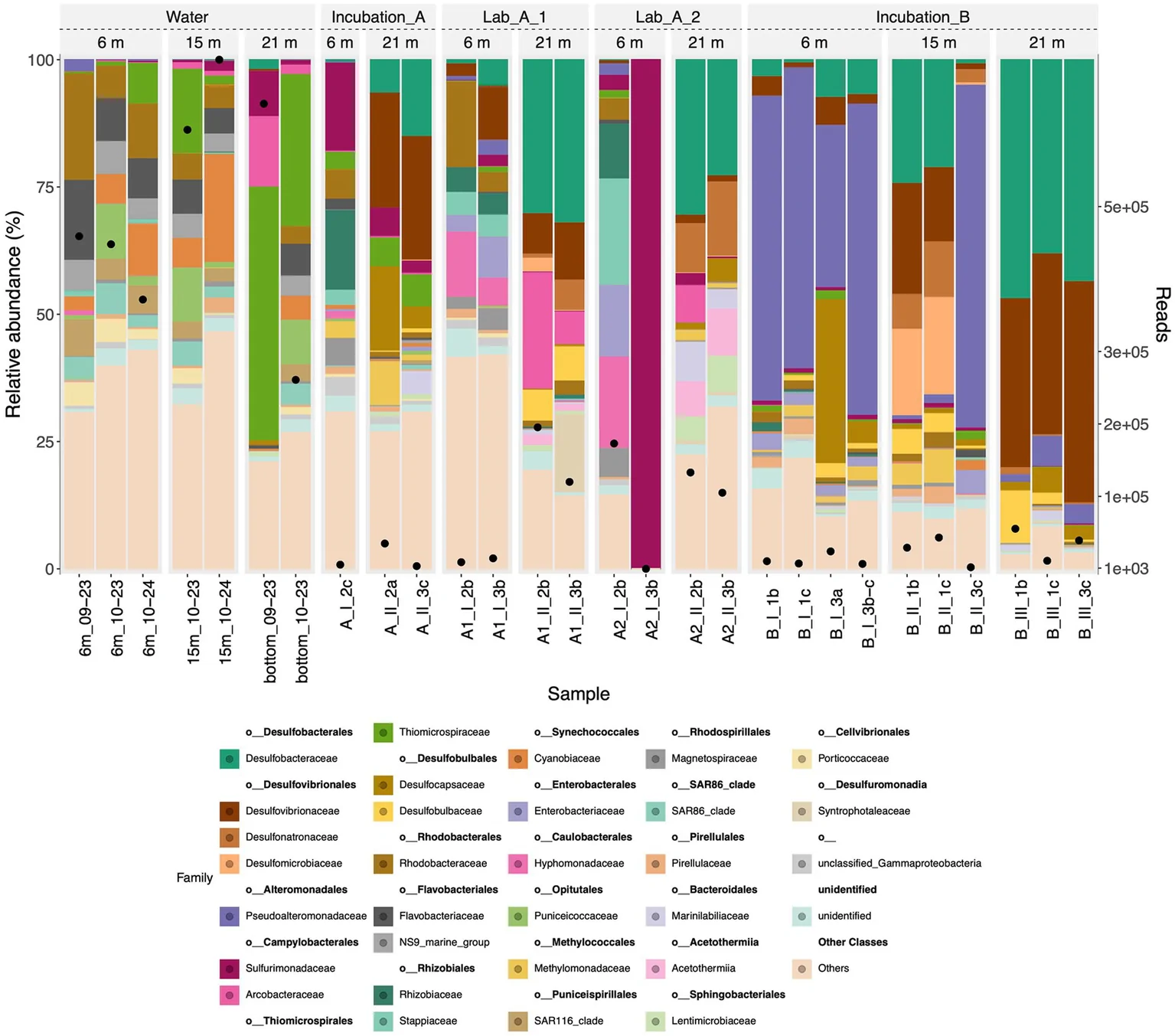
Taxonomic profiles of bacterial communities on family level. Shown are the 30 most abundant families across all analyzed samples. Black dots indicate the number of analyzed sequence reads of the respective sample. Sample names from the *in situ* and laboratory incubations follow the scheme introduced in Figure 1 with the addition of the number of the individual steel coupon.

The taxonomic profiles of the water column samples were highly diverse. They contained large proportions of low abundant bacteria, i.e., those not belonging to the 30 most abundant genera or families, in most of the samples (Figure 7; Supplementary Figure S3). The 6 m water column communities were dominated by aerobic (or facultative anaerobic), organotrophic bacteria typically found in marine environments. These comprised members of the *Rhodobacteraceae* (up to 20%), Flavobacteriales (up to 22%), *Cyanobiaceae* (up to 25%) and SAR116 clade (up to 7%). Being rather low abundant in the 6 m community in September 2023, *Puniceicoccaceae* peaked in October 2023 (11%) and *Thiomicrospiraceae* in October 2023 (8%). Other highly abundant bacteria in the September 2023 bottom water were members of the *Sulfurimonadaceae* (9%) and *Arcobacteraceae* (14%), which are known to also be involved in sulfide oxidation (Han and Perner, 2015; Roalkvam et al., 2015). The bottom water sample in October 2023 as well as the 15 m samples in October 2023 and 2024 appeared to represent mixed communities, combining the sulfide oxidizers of the stratified bottom water with the organotrophic surface communities in varying proportions (Figure 7).

#### *In situ* incubation A – 3 months (July to October 2023)

Where available, the individual parallels of the *in situ* incubated bacterial steel biofilm communities were in good agreement with each other (Figure 7). In the 3-month incubation A, the most highly abundant genera in the 6 m sample were *Sulfurimonas* (17%) and *Hoeflea* (14% on genus level, see Supplementary Figure S3). The latter has been described as oxidizing Fe(II), coupled to nitrate reduction (Laufer et al., 2016), making it a promising candidate for MIC inside the monopile. Furthermore, a relatively large proportion (6%) of magnetotactic, Fe-scavenging members of the *Magnetospiraceae* (Uebe and Schüler, 2016; Busigny et al., 2025) was observed (Figure 7). The 3-month incubation at 21 m water depth was characterized by high abundances (up to 49%) of SRB, such as *Desulfobacteraceae*, *Desulfovibrionaceae* and *Desulfocapsaceae* (Figure 7). Due to the production of highly corrosive H_2_S, these SRB are often seen as key players of MIC in anoxic environments (e.g., Xu et al., 2023).

#### Lab incubations A1 and A2

Interestingly, the bacterial diversity of the 6 m incubation (A) appeared to increase in the following lab incubations (A1), as the proportion of low abundant Bacteria increased. However, this effect was not observed when biofilm material from the *in situ* incubation was transferred to fresh steel coupons (incubation A2, see Figures 1, 7). One of the fresh steel coupon incubations (A2_I_3b) harbored only members of the *Sulfurimonadaceae*, coinciding with an overall low number of merged sequence reads (Figure 7). Compared to the 6 m *in situ* incubation steel sample (A_I_2c), an enrichment in organotrophic *Enterobacteriaceae* (up to 14%) and members of the *Hyphomonadaceae* (up to 18%) was observed in all other oxic laboratory incubations (Figure 7). The latter family harbors typical organotrophic primary biofilm producers frequently found in marine habitats (Langille and Weiner, 1998; Abraham and Rohde, 2014). The relatively high abundances of anaerobic SRB from the *Desulfobacteraceae* and *Desulfovibrionaceae* families (e. g. summing up to 16% in A1_I_3b) indicate the presence of anoxic niches in the oxic lab incubations of pre-incubated steel coupons (lab incubation A1, see Figure 7). The dominance of SRB in the bacterial communities of steel coupons incubated at 21 m water depth continued in the subsequent laboratory incubations – also when fresh steel coupons were used (lab incubations A1 and A2). However, abundance shifts of individual families were observed: while *Desulfovibrionaceae* and *Desulfocapsaceae* decreased (<24 and <17% *in situ* vs. up to <1 and <4% *ex situ*), the opposite trend became apparent for *Desulfobacteraceae* and *Desulfonatronaceae* (<15 and <0.1% *in situ* vs. <32 and <14% *ex situ*).

#### *In situ* incubation B (October 2023 to October 2024)

After 12 months of *in situ* incubation (incubation B), the bacterial communities were characterized by reduced numbers of low abundant families or genera (Figures 7; Supplementary Figure S3). Here, the 6 m incubations were vastly dominated by the genus *Pseudoalteromonas* (32% to 61% on genus level, see Supplementary Figure S3), which are known as heterotrophs ubiquitously found in marine environments with the potential of biofilm formation and antimicrobial functions as well as to influence biofouling and corrosion (Bosi et al., 2017; Jaume et al., 2025). Despite the presence of fully oxygenated seawater during the entire incubation period, a second highly abundant bacterial group in the 6 m incubation B comprised different sulfate reducing families, i. e. *Desulfobacteraceae*, *Desulfovibrionaceae*, *Desulfocapsaceae* and *Desulfobulbaceae* with the highest abundances in sample B_I_3a (summing up to 47%, see Figure 7). In turn, putatively sulfur-oxidizing representatives of *Sulfurimonadaceae* and *Thiomicrospiraceae* constituted 1 to 2% of the bacterial communities of the steel biofilms. One of the three analyzed steel coupons from 15 m water depth of incubation B exhibited a bacterial community resembling that from 6 m water depth, albeit with slightly smaller proportions of SRB (<6%, see Figure 7). The other replicates were dominated by various SRB of the orders Desulfobacterales, Desulfovibrionales and Desulfobulbales, adding up to 76% with the highest abundances of *Desulfomicrobiaceae* among all analyzed samples (up to19%, see Figure 7). The dominance of SRB was even greater at 21 m water depth in incubation B, where they constituted 81 to 93% of the bacterial community with *Desulfobacteraceae* (<47%) and *Desulfovibrionaceae* (43%) being the most abundant families (Figure 7).

### Community compositions of water column and steel biofilm communities – Archaea

**Figure 8:**
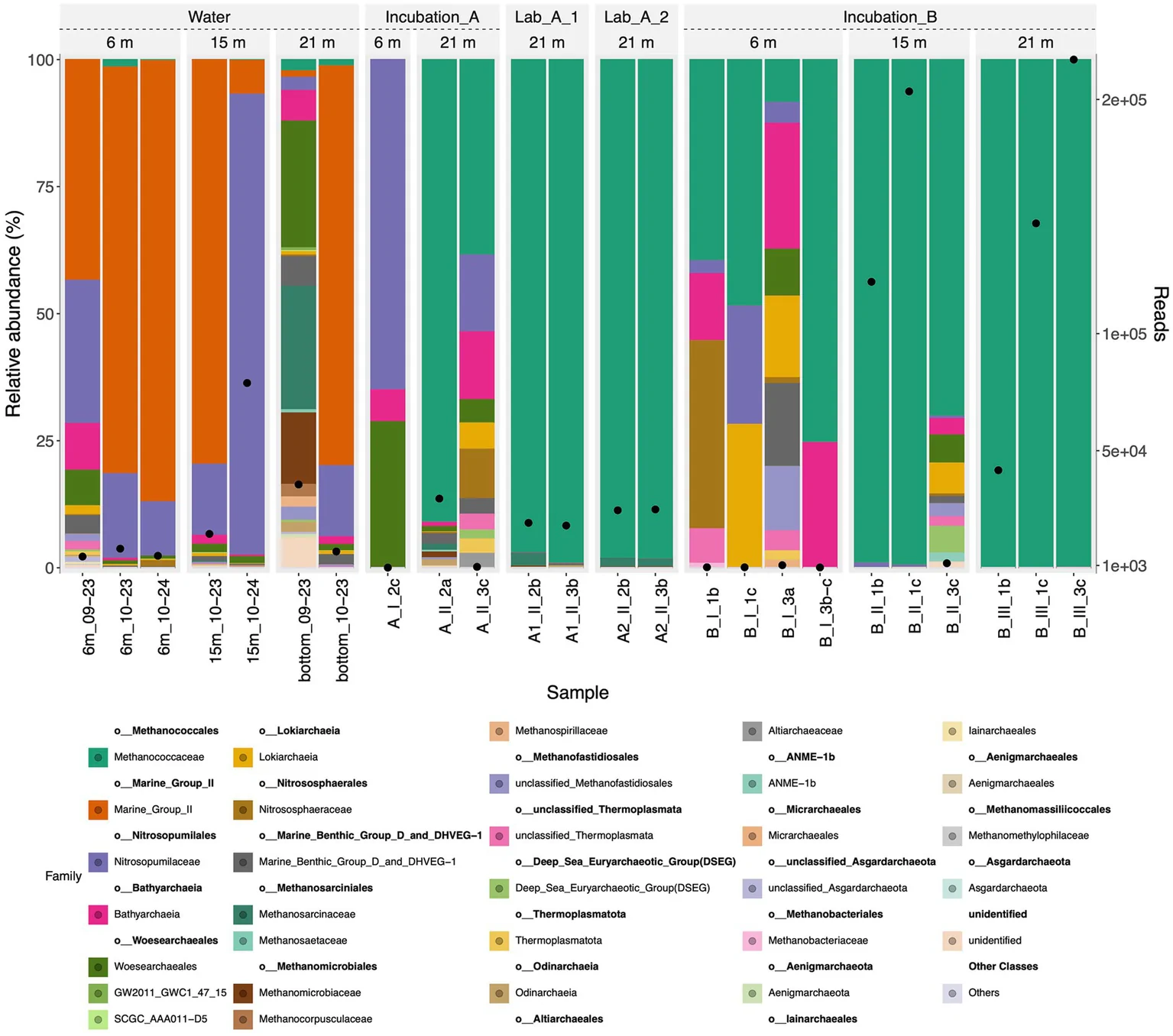
Taxonomic profiles of archaeal communities on family level. Shown are the 30 most abundant families across all analyzed samples. Black dots indicate the number of analyzed sequence reads of the respective sample. Sample names from the *in situ* and laboratory incubations follow the scheme introduced in Figure 1 with the addition of the number of the individual steel coupon.

Almost all of the archaeal water column communities were dominated by members of the family *Nitrosopumilaceae* (up to 90%) and/or Marine Group II (up to 87%, see Figures 8; Supplementary Figure S4), both of which are common colonizers of the open ocean – the latter being especially abundant in the photic zone (Zhang et al., 2015; Rinke et al., 2019; Santaro et al., 2019). Noticeable differences were observed in the water column samples from September 2023: these exhibited a higher diversity than those from the other time points. In addition to *Nitrosopumilaceae* and Marine Group II the 6 m sample contained relatively large proportions of Bathyarchaeia (9%) and Woesearchaeales (7%). The bottom water sample taken in September 2023 was characterized by high abundances of Woesearchaeales (25%), members of the Marine Benthic Group D and DHVEG-1 (6%), as well as methanogenic Archaea like *Methanosarcinaceae* and *Methanomicrobiaceae* (summing up to 46%, see Figure 8) (Buan, 2018). These anaerobic methanogens also prevailed in most *in situ* and lab incubations below 6 m water depth, peaking at 21 m in incubation B with 100% of the archaeal sequence reads. While no methanogens were detected in the 6 m steel biofilm of incubation A, the 6 m samples of incubation B contained up to 75% methanogens of the *Methanococcaceae* family and not further classified members of the Methanofastidiosales order (Figure 8). In incubation A, the archaeal community at 6 m water depth consisted only of three families: *Nitrosopumilaceae* (65%), unclassified Woesearchaeales (29%) and Bathyarchaeia (6%). These families were also highly abundant in one of the steel samples incubated at 21 m water depth (A_II_3c) with abundances ranging from 5% (Woeasearchaeales) to 15% (*Nitrosopumilaceae*). Members of the ammonia oxidizing *Nitrososphaeraceae* (10%) (Stieglmeier et al., 2014), Lokiarchaea (5%) and Marine Benthic Group D and DHVEG-1 (3%) were – in addition to the previously mentioned methanogens - among the other prevailing Archaea in this sample. Albeit in notably varying abundances and compositions across the parallels, members of the A_II_3c archaeal community were also found in high abundances (>3%) in the 6 m steel biofilms of incubation B (Figure 8). Only one of the samples incubated for 12 months below 6 m water depth harbored non-methanogenic Archaea at high abundances: in sample B_II_3c members of the uncultured Deep Sea Euryarchaeotic Group (5%), Lokiarchaeia (6%), Woesearchaeales (6%) and Bathyarchaeia (3%) accompanied the still dominating *Methanococcaceae* (70%, see Figure 8).

### Spearman correlations between distinct microbial lineages and water column properties

In order to identify environmental parameters influencing or being affected by the abundance and activity of distinct microbial lineages within the water column and steel biofilms inside the FINO3 monopile, Spearman correlation analyses were performed using the bacterial and archaeal community compositions and the water column properties (as shown in Figure 2) measured at the time of retrieval of the sample material (i.e., water samples or *in situ* incubated steel coupons). Due to the strongly differing community compositions, separate analyses were performed for the bacterial and archaeal water column and steel biofilm communities, respectively. No significant correlations between bacterial or archaeal lineages of the water column and the measured environmental parameters were found, which may be a result of the lower number of water samples compared to the number of analyzed steel biofilms.

**Figure 9:**
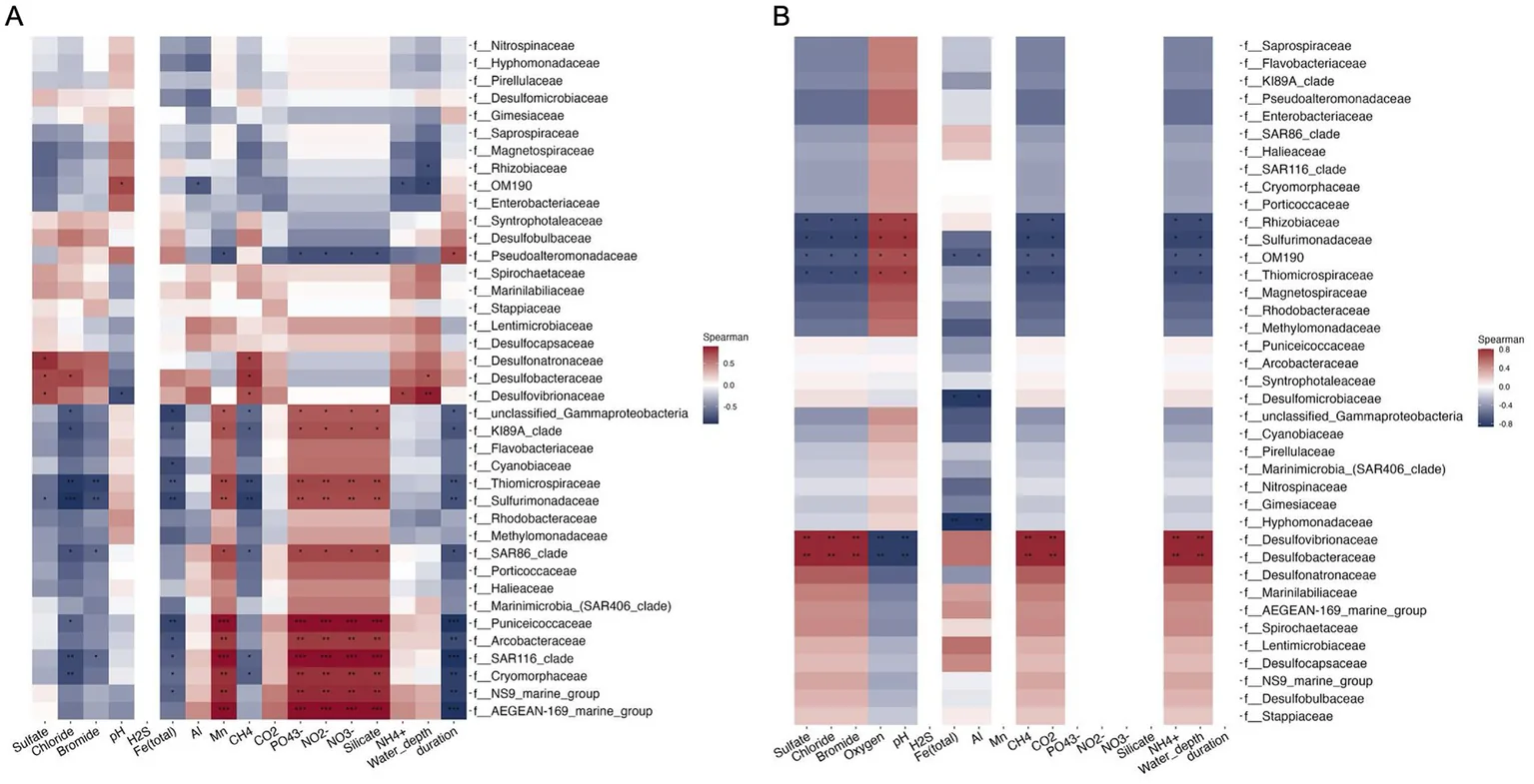
Heatmap of correlations between measured geochemical parameters of the water column and the 40 most abundant individual bacterial families of all *in situ* incubated steel biofilms **(A)** and only incubation B **(B)**. Oxygen was excluded from the correlation analysis with all steel samples, as it was only measured in 2024. Levels of significance are indicated by asterisks (* = *p* < 0.05, ** = *p* < 0.01, *** = *p* < 0.001).

Correlations between members of the *in situ* incubation communities and water column properties were further divided into two sets of analyses. The first consisted of both incubations but lacked oxygen as environmental parameter, as no oxygen measurements were available for the time of recovery of incubation A. The second only comprised the microbial communities of incubation B but also included oxygen. When analyzing all *in situ* incubated steel biofilms *Sulfurimonadaceae*, *Thiomicrospiraceae,* and not further classified *Gammaprotoebacteria* (all highly abundant in incubation A) showed significant positive correlations with manganese and most nutrients (PO_4_^3−^, NO_2_^−^, NO_3_^−^ and silicate). Significantly negative correlations were observed between these three families and measured anions (sulfate, bromide, chloride), total iron, and methane concentrations as well as the length of the incubation period (duration) (Figure 9A). The heterotrophic members of the *Pseudoalteromonadaceae* family (dominating in the 6 m and one of the 15 m biofilms of incubation B) were characterized by a significantly positive correlation with the incubation length (duration) and negative correlations with most nutrients as well as manganese concentrations (Figure 9A). The family *Rhizobiaceae*, which was highly abundant only in the 6 m sample of incubation A, showed a significant negative correlation with water depth. Significantly positive correlations with methane and sulfate concentrations were observed for the sulfate-reducing *Desulfobacteraceae*, *Desulfonatronaceae*, and *Desulfovibrionaceae*, which were dominating in most of the 15 m and 21 m steel biofilms of incubation B and (in case of the *Desulfovibrionaceae*) highly abundant in the 21 m incubation A (cf. Figures 7A, 9A). While *Desulfovibrionaceae* also significantly negatively correlated with pH and positively correlated with ammonium, *Desulfobacteraceae* showed a significantly positive correlation with bromide (in addition to methane and sulfate, see Figure 9A). If only incubation B is taken into account, *Desulfovibrionaceae* and *Desulfobacteraceae* both correlated negatively with oxygen and pH, while positive correlations were observed with sulfate, chloride, bromide, methane, CO_2_ and ammonium concentrations (Figure 9B). SRB of the *Desulfomicrobiaceae* (exclusively found in the 15 m incubation B with up to 19% abundance) and the low-abundant, heterotrophic *Hyphomonadaceae* and OM190 clade showed significantly negative correlations with total iron and aluminum contents (Figure 9B). The positive correlations of *Thiomicrospiraceae* and *Sulfurimonadaceae* with most nutrient-concentrations were not observed in the exclusive analysis of incubation B, resulting from the nutrient depletion observed in 2024. However, these families (together with *Rhizobiaceae* and clade OM190) were characterized by significantly positive correlations with oxygen and pH as well as negative correlations with the measured anions, methane-, CO_2−_ and ammonium- concentrations as well as with water depth (cf. Figures 9A,B).

All other bacterial families that correlated significantly with parts of the measured water column properties (e.g., *Puniceicoccaceae*, *Arcobacteraceae* and *Cryomorphaceae*) exhibited only low abundances (<1%) in the steel biofilm communities. However, the correlations of these families with nutrients (except ammonium), manganese concentrations and incubation duration exhibited the highest levels of significance observed in our correlation analysis (Figure 9A). The low abundance of these families in combination with the negative correlation of the incubation period suggests that these families were not an integral part of the steel biofilm community and thus strongly influenced by the water column properties.

**Figure 10:**
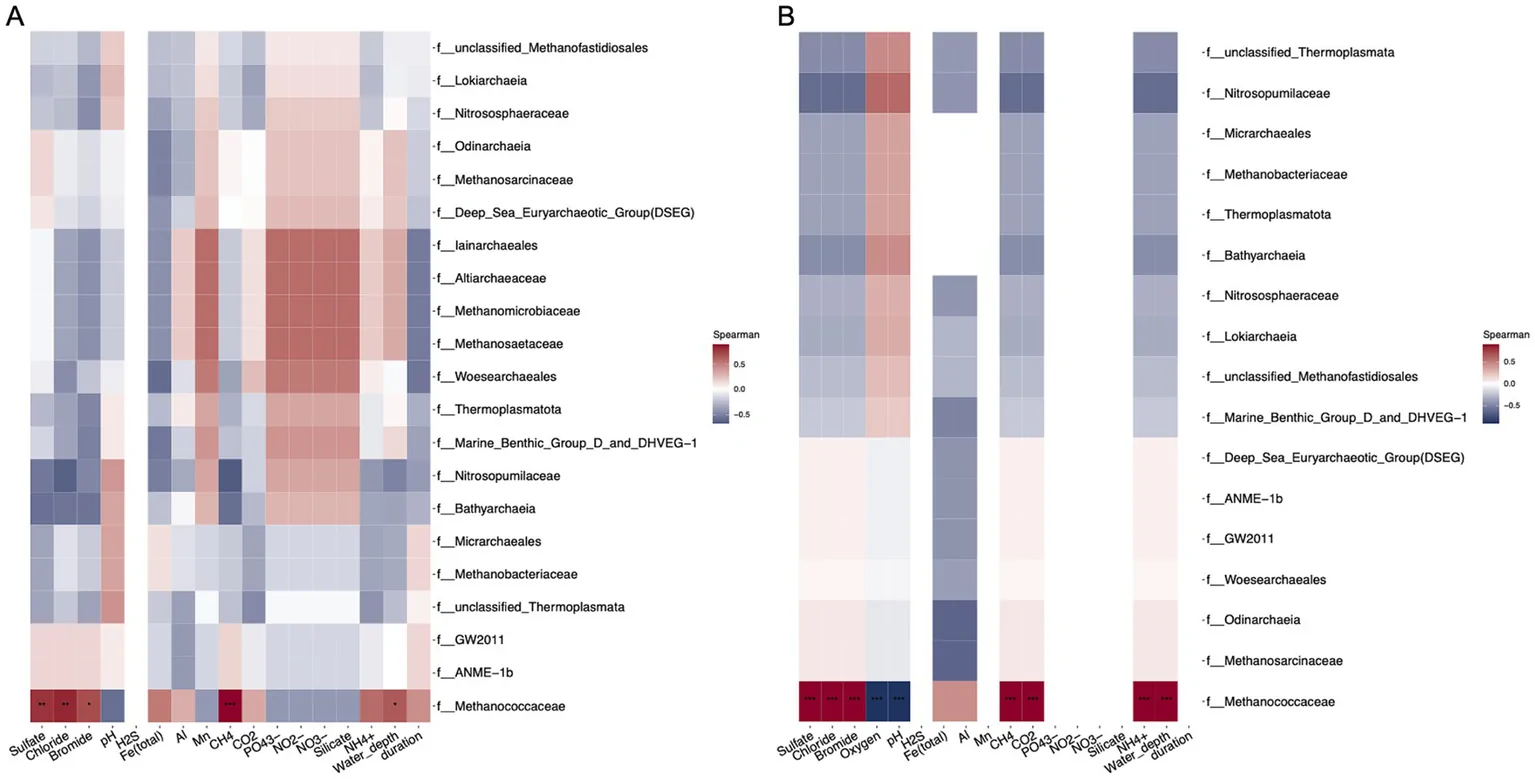
Heatmap of correlations between measured geochemical parameters of the water column and observed archaeal families of all *in situ* incubated steel biofilms **(A)** and only incubation B **(B)**. Oxygen was excluded from the correlation analysis with all steel samples, as it was only measured in 2024. Levels of significance are indicated by asterisks: (* = *p* < 0.05, ** = *p* < 0.01, *** = *p* < 0.001).

Among the archaeal steel biofilm communities only the methanogenic *Methanococcaceae* family, which was highly abundant in almost all incubations below 6 m water depth, showed significant correlations with at least some of the geochemical parameters measured in the water column of the FINO3 monopile. In the analysis of all incubations (omitting oxygen concentrations), *Methanococcaceae* correlated positively with methane and measured anion concentrations as well as water depth (Figure 10A). These correlations, albeit at an even higher level of significance, were also observed in the exclusive analysis of incubation B. Here, further significantly positive correlations with CO_2_ and ammonium concentrations as well as negative correlations with oxygen and pH levels were found (Figure 10B).

### Characterization of *in situ* incubated steel biofilms

For further characterization of the biofilms developed on the *in situ* incubated steel coupons, EPS extracts were obtained from corrosion products / biofilms scraped off the steel coupons. Due to limited sample sizes only one extraction per analyzed steel coupon could be performed. In the EPS extracts of incubation A proteins and neutral polysaccharides were below the respective limits of detection (Table 2). The concentrations of acidic polysaccharides increased with incubation depth, peaking at 519 μg/g wet biofilm (in the 21 m incubation A_II_2a). Uronic acid and lipid contents exhibited large deviations between the individual samples, thus no clear depth-related trend was visible (Table 2). However, with the peak at 868.2 μg/g wet biofilm the uronic acid contents in biofilms of incubation A were predominantly higher than those measured for incubation B, where they mostly remained below the limit of detection. The lipid levels, on the other hand, were more or less in the same range (2.0–5.0 mg/g of wet biofilm) for both incubations – with the single exception of sample A_I_2a (Table 2). In contrast to incubation A, neutral polysaccharides were measurable in all EPS extracts from incubation B, ranging from 15.7 to 75.7 μg/g of wet biofilm (Table 2). Acidic polysaccharides exhibited notable variations between the individual samples incubated at the same depth but were overall in the same range (274 to 546 μg/g of wet biofilm) as in incubation A (Table 2). Considerable amounts of proteins were only detected in biofilms of the 6 m and 21 m incubations B (but only in one of the two analyzed sample, each; see Table 2). Overall, the contents of the measured EPS components in both incubations were lower than expected. Apart from a limited matrix formation / low biofilm formation or a loss of biofilm material during the sampling process, this could also result from limitations of the method used for EPS extraction. A number of different EPS extraction methods has been published and evaluated, demonstrating differing efficiencies and drawbacks, often depending on the composition of biofilm and substrate. In order to avoid cell lysis and an overestimation of EPS contents or carryover of chemical compounds, we opted against chemical extraction procedures (e.g., using NaOH or formaldehyde) but used a physical method instead which may have led to an incomplete recovery of the EPS material (Comte et al., 2006; D’Abzac et al., 2010; Jachlewski et al., 2015). The fact that at least some of the measured parameters were more pronounced in the 12-month incubation B compared to the 3-month incubation A indicates that the biofilm in incubation B was more developed. However, given the (putative) limitations in sample size / number and EPS recovery as well as the analysis of only one timepoint (i.e., the end) of the incubations, the level of maturity and overall extent of the biofilms remains difficult to estimate.

**Table 2:** Measurements of EPS components extracted from *in situ* incubated S355 mild steel coupons.

Incubation	Incubation depth	Sample	Protein (μg/g)	Acidic polysaccharides (μg/g)	Neutral polysaccharides (μg/g)	Uronic acids (μg/g)	Lipids (mg/g)
Incubation A	6 m	A_I_2a	bdl	275.0	bdl	868.2	bdl
		A_I_3c	bdl	397.0	bdl	148.2	4.8
	21 m	A_II_2a	bdl	518.9	bdl	290.8	3.2
		A_II_3c	bdl	496.0	bdl	186.4	4.8
Incubation B	6 m	B_I_1b	32.4	417.1	48.6	bdl	2.0
		B_I_3c	bdl	260.4	15.7	202.4	4.0
	15 m	B_II_1b	bdl	439.0	52.9	214.4	3.0
		B_II_3c	bdl	517.0	71.4	bdl	4.0
	21 m	B_II_1b	bdl	274.1	37.1	bdl	5.0
		B_III_3c	54	546.0	75.7	bdl	4.0

Bdl = below detection limit (20 μg/g for proteins, 100 μg/g for acidic polysaccharides, 10 μg/g for neutral polysaccharides, 100 μg/g for uronic acids and 1 mg/g for lipids).

Further evidence for the microbial colonization of the incubated steel samples was gained by scanning electron microscopy. Sample material (either corrosion products or whole steel coupons) was fixed from all depths of both incubations and microbial cells and / or metabolites were found to be associated with corrosion products in all inspected samples (Figure 4; Supplementary Figure S5).

## Discussion

### Seasonally dynamic conditions shape offshore foundation ecosystems

The inside of the FINO3 foundation structure is a largely enclosed, dynamically stratified marine environment, which episodically shifts between oxygenated and oxygen-depleted states. Stratification in the summer months causes strong vertical redox gradients below 15 m water depth, where anoxic, reduced conditions can develop. This allows for sulfide, methane CO_2_, iron and aluminum to accumulate in bottom waters, likely persisting for several weeks, as indicated by our measurements of geochemical water column properties (cf. Figure 2). Stratification in the foundation’s interior becomes disrupted during storm-driven mixing events, causing re-ventilation of bottom waters and oxidation of reduced compounds. Compared to recent surveys of nutrient contents in the North Sea and especially in the German Bight (Siems et al., 2024) our measurements showed highly elevated peak ammonium concentrations inside the FINO3 monopile, exceeding the typical seawater concentrations by up to a factor of 1,000. The peak concentrations of the other nutrients were also higher than those typically measured in the German Bight but the effect was less pronounced as for ammonium (cf. Figure 2; BSH, 2016; Siems et al., 2024). The iron concentrations measured inside the monopile were at least 50-fold and up to 400-fold higher than the median iron concentrations in the German Bight (Siems et al., 2024), which likely indicates corrosion related iron accumulation inside the steel structure, especially during the seasonal stratification.

Overall, the measured geochemical data and identified microbial taxa in the water column (as well as steel biofilms) suggest intense microbial cycling of carbon, sulfur, and nitrogen, depending on the redox status of the (stratified) water body. Even though no significant Spearman correlations were observed between members of the microbial communities of water samples and the measured geochemical parameters at the respective water depths, notable differences in the community compositions during different stratification stages were observed in bottom waters. For example, the highest abundances of *Thiomicrospiraceae* and *Sulfurimonadaceae,* known to oxidize sulfide and/or hydrogen (and iron in the case of *Thiomicrospiraceae*) were detected in bottom waters of the FINO3 monopile in September 2023, when sulfide accumulations and thus the highest substrate abundance for SOB were observed (cf. Figures 2, 7; Han and Perner, 2015; Hansen and Perner, 2016; Barco et al., 2017; Laufer-Meiser et al., 2024). Given the absence of oxygen and nitrate in bottom waters in September 2023 (Figure 2), the main electron acceptors of these families were depleted at the time of sampling. Thus, the abundant *Thiomicrospiraceae* and *Sulfurimonadaceae* members may represent declining populations of SOB. Since our 16S rRNA gene analyses are based on RNA extractions targeting the active microbial communities, however, it is more likely that the observed SOB were using alternative electron acceptors. Both nitrite and Mn were detected in bottom waters of September 2023 (Figure 2) and have previously been reported as alternative electron acceptors for members of the *Sulfurimonadaceae* and *Thiomicrospiraceae* (only nitrite) families (Han and Perner, 2015; Henkel et al., 2021; Liu X. et al., 2021). The high abundance of *Thiomicrospiraceae* in the 6 m water sample in the following month (October 2023) most likely results from the storm-related mixing of the water column as also indicated by the geochemical profiles (cf. Figure 2).

Typically, sulfidic bottom waters develop under anoxic conditions when sulfide (resulting from SRB activity) is released from marine sediments (Perner et al., 2022; Alampalli et al., n.d). The *in situ* incubations of steel coupons (exhibiting high abundances of SRB, see Figure 7) suggest that inside the monopile SRB colonizing the steel walls of the structure may provide an additional source for sulfide. Likewise, methanogenic Archaea produce methane in anoxic sediments which (if not fully removed by methanotrophic microorganisms) can emit from the sediment (Reeburgh, 2007). Again, methanogenic archaea colonizing the monopile walls may further contribute to methane levels in the water column, as suggested by high abundances in the archaeal steel incubation communities (Figure 8). Furthermore, and in contrast to SRB, methanogens appear to thrive in the bottom water of the monopile under stratified conditions, as they constitute 48% of the archaeal community in the analyzed water sample of September 2023. SRB and methanogens compete over common electron donors (i.e., hydrogen, acetate and methylated compounds) and due to higher substrate affinities, SRB are often thought to win this competition (Oremland and Polcin, 1982). However, as also noticed by co-colonization of SRB and methanogens and the positive correlation of SRB with CH_4_ in our steel incubations, the co-existence has already been shown before in isolated environments and incubation studies (Stams et al., 2005; Sela-Adler et al., 2017).

### Potential for MIC and underlying processes inside unprotected monopiles

**Figure 11:**
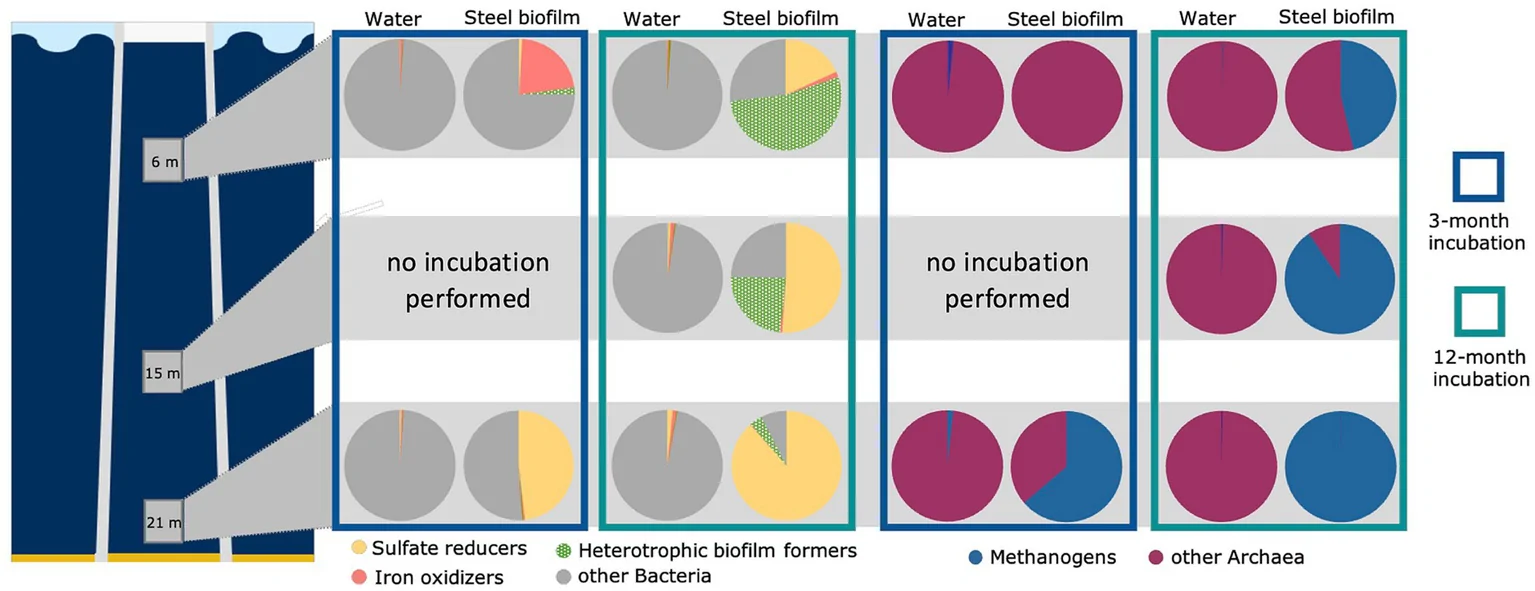
Distribution of microbial groups typically involved in MIC. The displayed pie charts summarize the abundances of typical representatives of the respective physiological traits, based on the taxonomic assignments of the 16S rRNA gene analyses.

In all of the analyzed *in situ* incubated steel biofilms, microbial taxa commonly associated with MIC were identified with abundances of up to 93% (of the bacterial communities) and 100% (of the archaeal communities), respectively (Figure 11). Still, the presence of MIC-associated microbes on a visibly corroded surface itself does not provide sufficient evidence that MIC actually occurred during the *in situ* incubation inside the monopile. The discrimination between electrochemical and microbially influenced corrosion and thus the identification of MIC in the field is a commonly acknowledged challenge (Knisz et al., 2023; Xu et al., 2023). The use of sterile / abiotic controls to evaluate the pure electrochemical corrosion to subtract it from the total corrosion, is only feasible under laboratory conditions. Yet, such studies are only suitable to investigate the MIC potential and influence of cultivable microorganisms under narrowly defined conditions. Thus, they may not reflect the full extent of MIC in a dynamic environment, as again demonstrated by the community shifts in subsequent laboratory incubations following incubation A in this study. Further indications for MIC formation during the *in situ* incubations inside the FINO3 monopile result from the visual observations and EDS measurements of incubated steel coupons. Indications for the presence of metabolic products typically produced by MIC associated microorganisms and associated with MIC processes were found on all analyzed steel samples. These comprise stalk and sheath structures identified in the SEM images (indicative of metabolites of Fe-oxidizing bacteria) as well as indications for the presence of FeS in incubation B and the 21 m incubation A. Even though our experimental data do not provide a direct evidence for the presence of FeS on incubated steel coupons, the elemental compositions of corrosion products (EDS results), characteristic sulfidic smell of the recovered steel coupons, measured (episodic) accumulation of H_2_S in bottom waters inside the monopile, and presence of SRB during the *in situ* incubations, let it appear highly likely that FeS is the dominating corrosion product in the bottom water incubations of S355 mild steel. The strongest indication for active MIC processes however, results from the pitting observed in incubation B. In contrast to stainless steel, pitting is an atypical corrosion behavior for electrochemical corrosion of S355 mild steel and thus may indicate a microbial influence (Starosvetsky et al., 2001; Ryan et al., 2002). Electrochemical studies like open-circuit potential (OCP) monitoring, electrochemical impedance spectroscopy (EIS), and/or electrochemical frequency modulation analyses may provide further insights into the corrosion characteristics, aiding in the discrimination between electrochemical and microbially influenced corrosion (Abilio et al., 2026; Correa et al., 2026). Yet, there is currently no in-field solution available for these methods (Abilio et al., 2026) and thus the respective electrochemical monitoring during our *in situ* incubations was not possible.

Among the microbial groups commonly involved in MIC, SRB and methanogens exhibited the highest abundances in steel biofilms, especially under anoxic conditions (21 m water depth) and/or after prolonged incubation (12-month incubation B). SRB are primarily associated with indirect MIC, enhancing corrosion through the production of highly corrosive sulfide and leading to the formation of iron sulfides (FeS). However, a limited number of lithotrophic (hydrogenotrophic) SRB of the *Desulfobulbaceae* and *Desulfovibrionaceae* demonstrated the capability of direct extracellular electron transfer from Fe^0^ (eMIC), based on specialized cytochromes (Enning and Garrelfs, 2014 and references therein). In contrast to SRB, methanogens do not produce corrosive metabolites. Here, eMIC represents the only MIC mechanism and has been predominantly observed in certain hydrogenotrophic methanogens – including representatives of the *Methanococcaceae* family. It may involve extracellular hydrogenase enzymes which generate hydrogen from electrons derived from Fe^0^ as an intermediate, which is then used for methanogenesis (Tsurumaru et al., 2018; Kleinbub et al., 2025). Although both, SRB and methanogens, are commonly described as strict anaerobes and correlated negatively with oxygen in the Spearman correlation analyses, they were also found in steel biofilms at shallower water depths in incubation B, characterized by fully oxic conditions of the surrounding water over the whole incubation period. This suggests the presence of micro-scale anoxia in the respective biofilms, enhancing the potential for persistent damage caused by MIC. Furthermore, this observation supports the hypothesis of MIC-related accumulation of dissolved Fe and biogenic CH_4_ indicated by water column properties measured in October 2023 inside the FINO3 monopile (see above). In the 6 m (and in one replicate of the 15 m) incubation B, this putative MIC effect may either be counter balanced or enhanced by the dominance of *Pseudoalteromonadaceae*, which have been described as either passivating steel surfaces against MIC or accelerating MIC in steel incubations (Moradi et al., 2014; Jaume et al., 2025). Based on the lesser surface roughness of steel coupons from the 6 m incubation B (compared to the 6 m incubation A, cf. Figure 5), a passivating effect of the *Pseudoalteromonadaceae* biofilm appears more likely in our case. *Pseudoalteromonadaceae* abundances were negligible in incubation A, instead high abundances of *Hoeflea* were found in the 6 m incubation. Given the previously reported Fe-oxidation and nitrate-reduction potential of *Hoeflea* strains, it may play a role in potential MIC processes inside the FINO3 monopile (Sorokina et al., 2012; Laufer et al., 2016; Warashina et al., 2024). The corrosion potential of nitrate-depending Fe-oxidation would likely be particularly high, as the highly reactive metabolic intermediate nitrite is formed, which can also oxidize Fe (Liu B. et al., 2021; Xu et al., 2023). Notable abundances of active members of bacterial families known to be involved in Fe-oxidation or -incorporation (i.e., *Hyphomonadaceae*, *Mariprofundaceae* and *Magnetospiraceae*) were also limited to incubation A but summing up to 24% in the 6 m incubation A (Figure 10). Since no significant correlations with environmental parameters were observed for these families, we hypothesize that the Fe-oxidizers represent a part of the initial steel colonizers as already observed in other steel incubation studies (Emerson, 2018 and references therein). Likely, later on in the course of the 12-month incubation B, they were suppressed by the heterotrophic biofilm-forming *Pseudoalteromonadaceae*. Both, Fe-oxidizers and the facultative aerobic *Pseudoalteromonadaceae* likely contribute to oxygen depletion and the formation of anoxic niches, facilitating the colonization with anaerobes like SRB and methanogens in the presence of fully oxygenated seawater (cf. Alonso and Pernthaler, 2005; Xu et al., 2023). Similar succession patterns in MIC biofilms, especially resulting in the enrichment of SRB have also been reported from laboratory incubation studies inoculated with environmental samples, albeit over a shorter time period (Nolan et al., 2025). In combination with the correlation analyses between measured geochemical water column parameters and observed microbial taxa, this succession indicates a greater influence of environmental factors on the microbial community composition in the initial phase of the studied steel biofilms. Most correlations were observed with bacterial families that were highly abundant in the shorter incubation A and the presence of anaerobic microorganisms in incubation A was limited to steel coupons incubated at a water depth where anoxia (presumably) prevailed over the whole incubation period. The significantly positive correlation of *Thiomicrospiraceae* and *Sulfurimonadaceae* (highly abundant only in steel biofilms of incubation A) with nutrient concentrations and their depletion at the end of incubation B indicate that the nutrient availability may also have an impact on a more mature steel biofilm. However, other factors such as antimicrobial metabolites secreted by the dominating *Pseudoalteromonas* may also have affected the microbial community composition and *Thiomicrospiraceae* / *Sulfurimonadaceae* abundance (Offret et al., 2016). Further complex interactions are taking place in (MIC) biofilms, including the establishment of micro-scale niches, metabolite-cycling and inter-species electron transfer (Gu et al., 2021; Wang et al., 2026). This complicates the determination of external influences like the water column geochemistry, especially in highly dynamic environments such as the FINO3 monopile.

Whether the cathodic corrosion protection systems installed inside the FINO3 monopile have an influence on biofilm formation and potential MIC processes on S355 steel surfaces also remains an open question. Both systems (impressed current and the sacrificial aluminum anode) need an electrical connection to the steel structure being protected in order to provide the cathodic reaction and corresponding corrosion protection (Baeckmann et al., 1997). Yet, none of the steel coupons incubated and analyzed within this study were connected to either of the two systems, ruling out a direct influence of these corrosion protection measures. Aluminum ions released from the sacrificial anodes might affect the microbial steel biofilm communities inside the FINO3 monopile as suggested by negative correlations of aluminum with *Desulfomicrobiaceae*, *Hyphomonadaceae*, and the OM190 clade. However, the measured concentrations of dissolved aluminum (even though elevated compared to ambient seawater) still remain below inhibitory concentrations reported in the literature (Piña and Cervantes, 1996; Buvignier et al., 2019). Thus, the presented results display a baseline study of the potential for microbial steel colonization and putative MIC inside unprotected monopile structures. Further studies investigating the influence of cathodic corrosion protection systems will need to follow.

## Conclusion

Altogether, the presented 16S rRNA gene analyses, steel biofilm characterizations, and corroded steel surface analyses give strong indication that the occurrence of MIC inside unprotected monopile structures is highly likely. First, we found high abundances of microbial taxa typically associated with MIC (i.e., Fe-oxidizers, biofilm forming microbes, SRB and methanogens) colonizing the incubated mild steel coupons, especially when compared to water column communities of the respective water depths (Figure 11). Second, indications for the presence of microbial precipitates (e. g. in form of twisted stalks) and corrosion products uncommon for electrochemical corrosion (i.e., the likely presence of FeS) as well as the observed pitting on corroded steel surfaces give further indications for the potential onset of different MIC processes during the *in situ* exposure of unprotected mild steel, intensifying with exposure time. In addition to the (initial) substrate availability and environmental conditions, the duration of the exposure and thus the maturity of the steel biofilm appears to influence the steel colonization, community composition and respective processes. Furthermore, the present study demonstrates that a monopile structure, as commonly used as foundations for maritime offshore infrastructure, is a biogeochemically active reactor where – depending on the respective construction - restricted water exchange, seasonal water column stratification and steel colonization creates hotspots for anaerobic and aerobic microbial processes, which likely also influence microbially influenced as well as electrochemical corrosion. Hence, interiors of offshore wind turbine steel-based foundations are (biogeo-) chemically active structures hosting dynamic ecosystems, affecting material degradation and infrastructure lifespan.

## Data Availability

Raw reads of the 16S amplicon sequencing were deposited in the sequence read archive (SRA) of the National Center for Biotechnology Information (NCBI) and can be found under project number PRJNA1462358. Data of geochemical measurements inside the monopile can be found in the PANGAEA database (https://doi.pangaea.de/10.1594/PANGAEA.995830 and https://doi.pangaea.de/10.1594/PANGAEA.995835).

## Footnotes

1
https://www.fino3.de/en/
