# Supplementary material for: Seasonally dynamic ecosystems within offshore wind power monopile foundations support microbially influenced corrosion
Source: Front Microbiol. 2026 Sep 3;17:1886489. doi: 10.3389/fmicb.2026.1886489 (PMC13582271; doi:10.3389/fmicb.2026.1886489)
Supplement: Supplementary file 1 [file Data_Sheet_1.PDF]

Supplementary information for:

The interiors of offshore wind power monopile foundations are seasonally dynamic ecosystems that support microbially influenced corrosion

Adam-Beyer, N.[*], Skottke, C.[2], Schmidt, M.[3], Solterbeck, C.-H.[4], Wyler, E.[5], Landthaler, M.[5,6], Schloesser, J.[4] & M. Perner[1*]

[1] Geomicrobiology, GEOMAR Helmholtz Centre for Ocean Research Kiel, Kiel, Germany
[2] F&E GmbH, Kiel, Germany
[3] Benthic Biogeochemistry, GEOMAR Helmholtz Centre for Ocean Research Kiel, Kiel, Germany
[4] University of Applied Sciences Kiel, Kiel, Germany
[5] Max Delbrück Center for Molecular Medicine in the Helmholtz Association, Berlin Institute for Medical Systems Biology (BIMSB), Berlin, Germany
[6] Institute for Biology, Humboldt University of Berlin, Berlin, Germany

This file contains:
Supplementary figures S1 to S5
Supplementary table S1

Figure S1: Location of the FINO3 monopile in the German North Sea

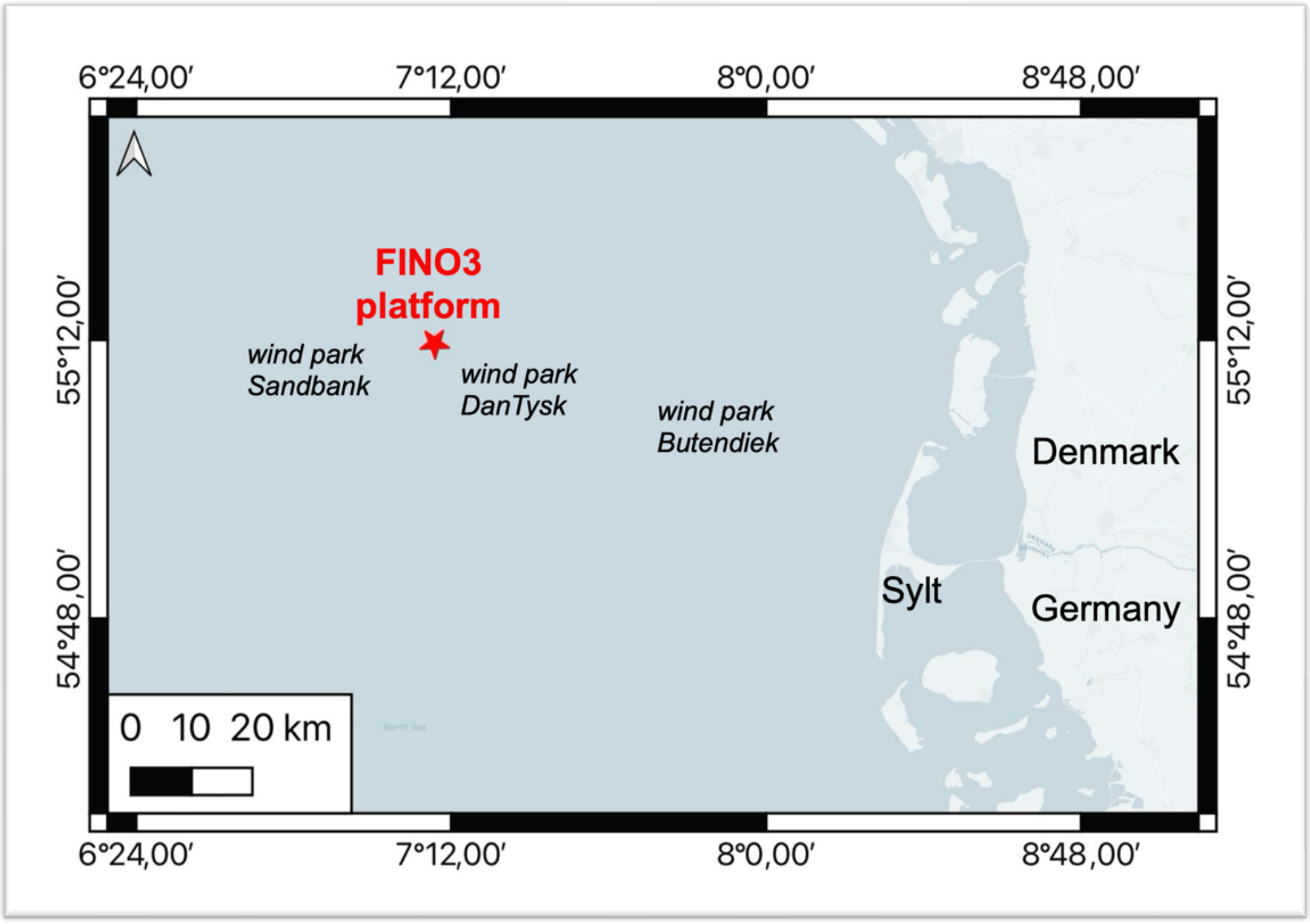

Figure S2: Shannon diversity indices of bacterial (A) and archaeal (B) 16S metabarcoding. Asterisks indicate the level of significance in the difference between the individual sample groups. ns = not significant

A

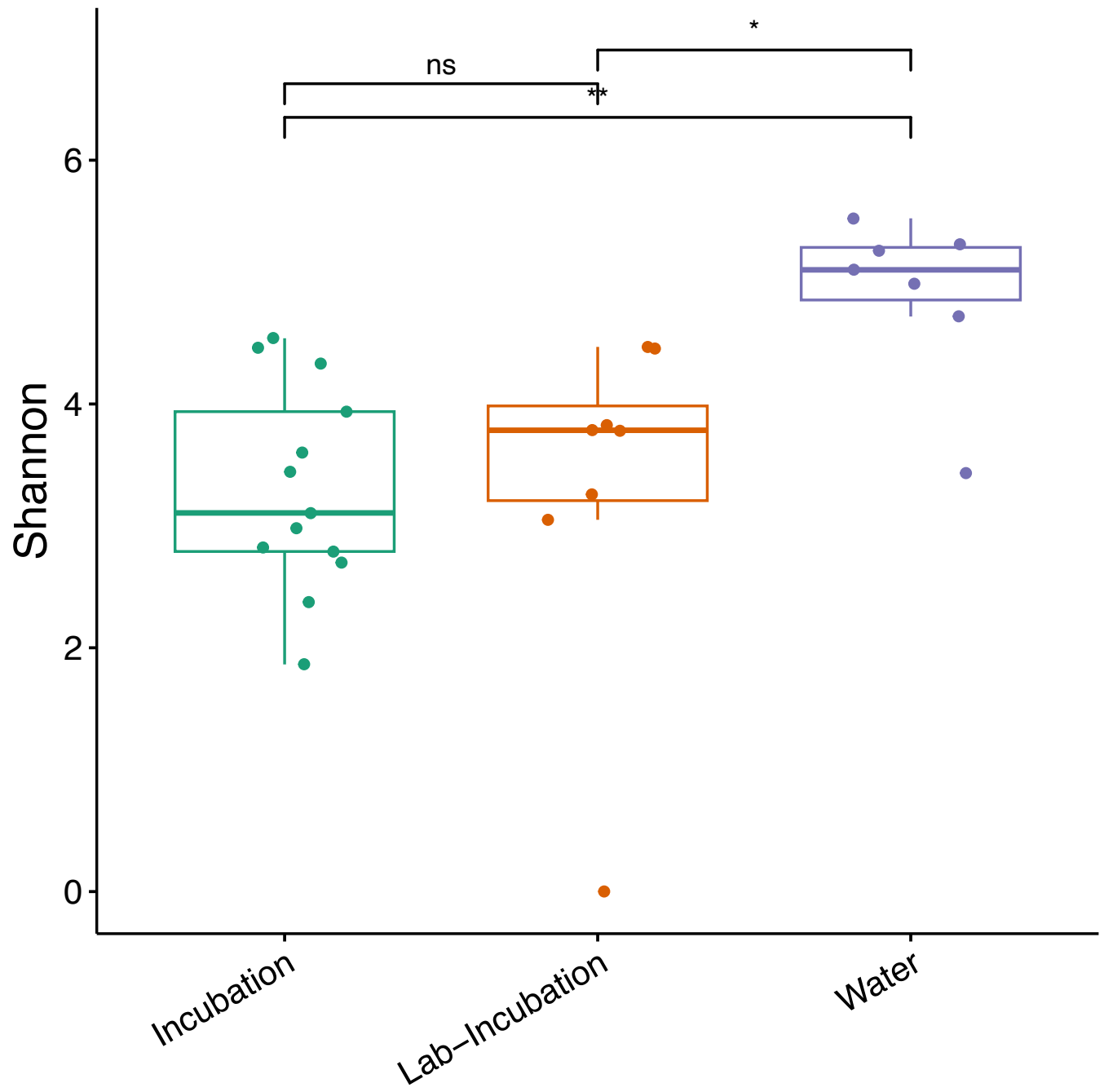

B

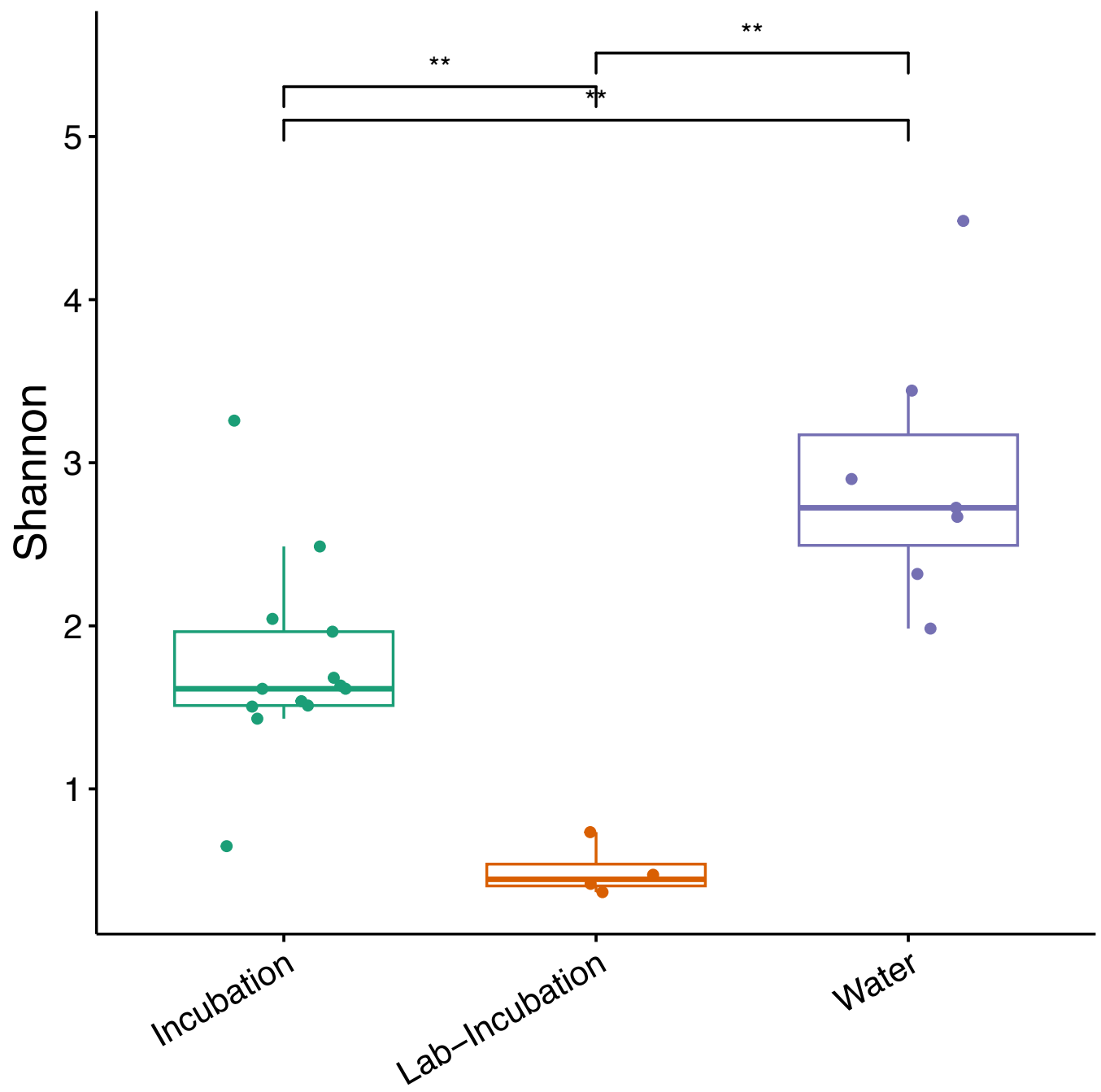

Figure S3: Taxonomic profiles of bacterial communities on genus level. Shown are the 30 most abundant genera across all analyzed samples. Black dots indicate the number of analyzed sequence reads of the respective sample.

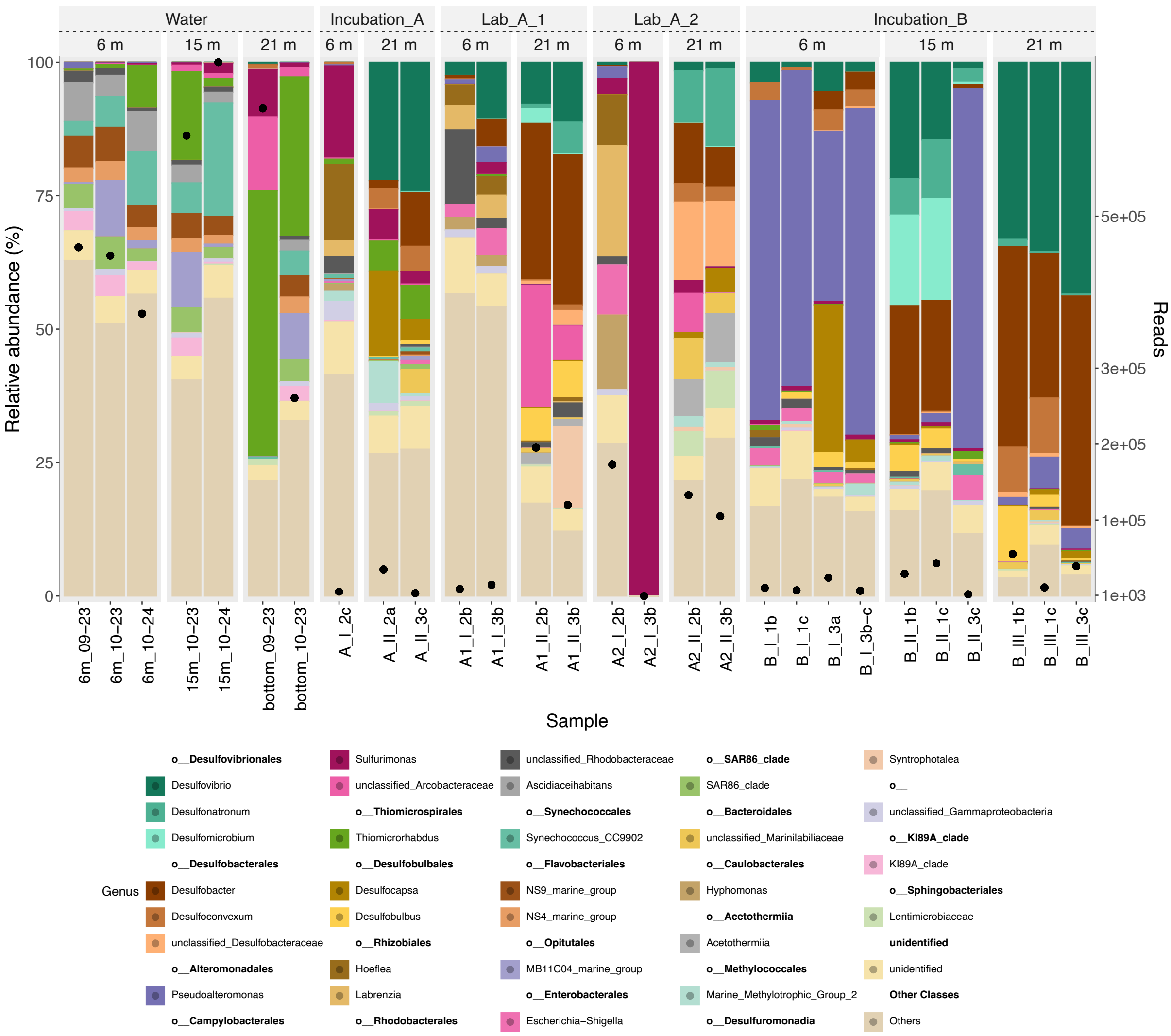

Figure S4: Taxonomic profiles of archaeal communities on genus level. Shown are the 30 most abundant genera across all analyzed samples. Black dots indicate the number of analyzed sequence reads of the respective sample.

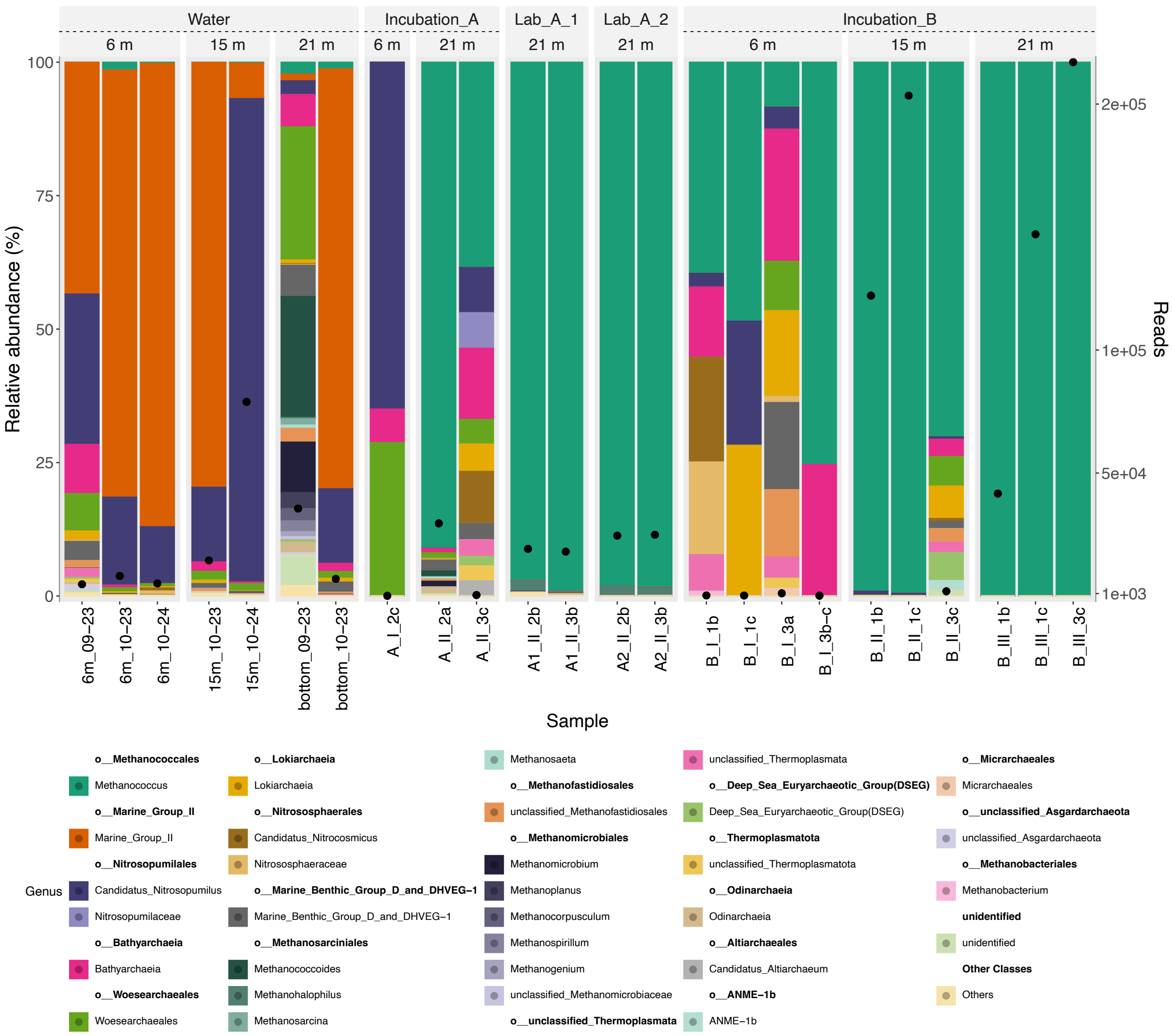

Figure S5: SEM images of incubated steel coupon surfaces (A, C, E, G, and I) and fixed corrosion products (B, D, F, H, and J) of incubation A (A-B, G-H) and incubation B (C-F, I-J).

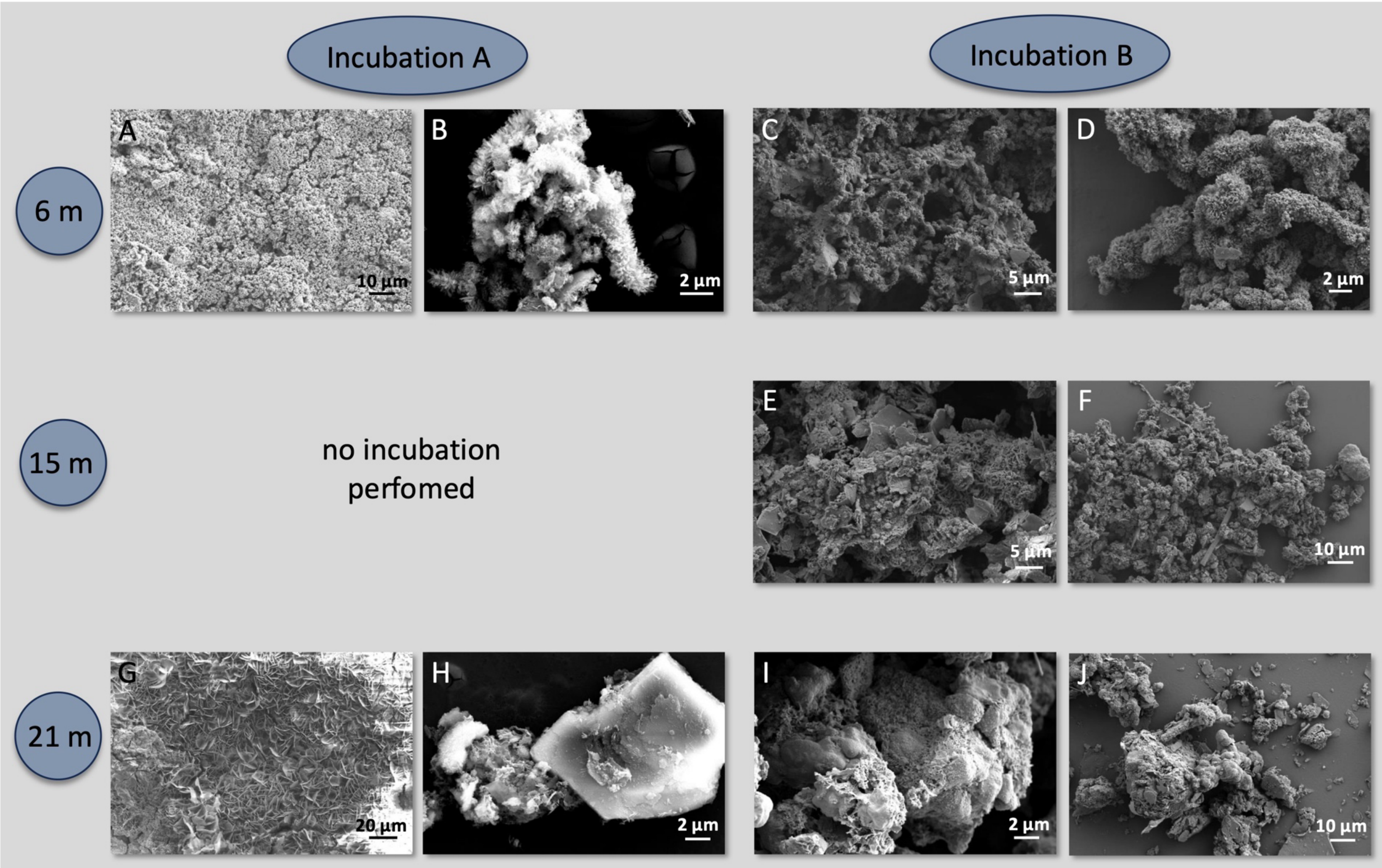

Table S1: Overview of analyzed water samples and in situ incubated S355 mild steel coupons. Performed analyses of the individual samples are indicated by a + (analysis performed) or – (analysis not performed).

Sample type	Sample name	Water depth	Incubation id	Incubation type	Incubation time	Sample retrieval / end of incubation	Remark	16S rRNA gene profiling	EPS analyses	SEM	EDS	3D microscopy
Water column	6m_09-23	6 m	---	---	---	September 2023	-	+	-	-	-	-
	bottom_09-23	21 m	---	---	---		-	+	-	-	-	-
	6m_10-23	6 m	---	---	---	October 2023	-	+	-	-	-	-
	15m_10-23	15 m	---	---	---		-	+	-	-	-	-
	bottom_10-23	21 m	---	---	---		-	+	-	-	-	-
	6m_10-24	6 m	---	---	---	October 2024	-	+	-	-	-	-
	15m_10-24	15 m	---	---	---		-	+	-	-	-	-
Corroded steel	A_I_1a	6 m	A	in situ	3 months	October 2023	-	-	-	+	-	-
	A_I_2a						-	-	+	-	-	-
	A_I_2b						-	-	-	+	+	+
	A_I_2c						-	+	-	-	-	-
	A_I_3c						-	-	+	+	-	-
	A_II_1a	21 m					-	-	-	+	+	+
	A_II_1c						-	-	-	+	-	-
	A_II_2a						-	+	+	+	-	-
	A_II_3c						-	+	+	-	-	-
	A1_I_2b	6 m	A1	laboratory	3 months in situ +3 months laboratory	January 2024	pre-incubated steel	+	-	-	-	-
	A1_I_3b							+	-	-	-	-
	A1_II_2b	21 m						+	-	-	-	-
	A1_II_3b							+	-	-	-	-

	A2_I_3b	6 m	A2		3 months		fresh steel with inoculum from inc. A	+	-	-	-	-
	A2_I_2b							+	-	-	-	-
	A2_II_2b	21 m						+	-	-	-	-
	A2_II_3b							+	-	-	-	-
	B_I_1b	6 m	B	in situ	12 months	October 2024	-	+	+	-	-	-
	B_I_1c						-	+	-	+	-	+
	B_I_2b						-	-	-	+	+	-
	B_I_3a						-	+	-	-	-	-
	B_I_3b-c						-	+	+	-	-	-
	B_II_1b	15 m					-	+	+	-	-	---
	B_II_1c						-	+	-	+	-	+
	B_II_2b						-	-	-	+	+	---
	B_II_3c						-	+	+	-	-	---
	B_III_1b	21 m					-	+	+	-	-	---
	B_III_1c						-	+	-	+	-	+
	B_III_2b						-	-	-	+	+	---
	B_III_3c						-	+	+	-	-	---
